# A tale of two management programs: Insights from a state-line wildlife disease outbreak

**DOI:** 10.1093/pnasnexus/pgaf387

**Published:** 2025-12-10

**Authors:** Andrew B Whetten, Trevor J Hefley, Christopher N Jacques, Daniel J Storm, Daniel P Walsh

**Affiliations:** Avian Population Studies, Cornell Lab of Ornithology, 159 Sapsucker Woods Rd, Ithaca, NY 14850, USA; Department of Statistics, Kansas State University, 1116 Mid-Campus Dr North, Manhattan, KS 66506, USA; Department of Statistics, Kansas State University, 1116 Mid-Campus Dr North, Manhattan, KS 66506, USA; Illinois Department of Natural Resources, 1 Natural Resources Way, Springfield, IL 62702, USA; Wisconsin Department of Natural Resources, 101 S Webster St, Madison, WI 53707, USA; U.S. Geological Survey, Montana Cooperative Wildlife Research Unit, Wildlife Biology Program, University of Montana, Missoula, MT 59812, USA

**Keywords:** chronic wasting disease, machine learning, social–ecological systems, white-tailed deer, wildlife management

## Abstract

Response to infectious disease outbreaks and ongoing management actions can vary greatly across geopolitical units. Understanding relationships between management actions of governance systems within social ecological systems (SES) is important when resource units (e.g. wildlife) and users (e.g. humans) inextricably connect them. Surveillance efforts provide critical information to governance systems for diseases such as coronavirus, influenza, and ebola. However, diseases that spread rapidly across multiple SESs are coarsely managed and monitored over weeks, months, or years. Such time and spatial constraints can challenge evaluation of management actions. Assessing importance of disease management across SESs for slow-moving infectious diseases can provide rare insights about management actions. We investigated an exemplary outbreak of Chronic Wasting Disease (CWD) in white-tailed deer populations along the geopolitical border of Illinois and Wisconsin, USA. We illustrated challenges of disease management in wildlife populations along geopolitical borders, where differences in management actions of adjacent governance systems were stark and subject to sudden change. Our analysis provided evidence of abrupt change in outbreak progression following drastic changes in management actions. In addition, we showed evidence of inconsistent and highly variable outcomes of management actions along a geopolitical border when adjacent governance systems are unable to cooperatively manage across interconnected SESs. Total annual harvest in a county is a primary management action used to control CWD, where increasing harvest potentially reduces prevalence. We showed that the effects of increasing total county-level harvest on cumulative incidence of CWD were highly heterogeneous. In some counties, increasing harvest had the opposite of the intended effect.

Significance Statement Evaluating the response of adjacent governance systems to an infectious disease outbreak provides the potential to understand practical challenges of disease management across geopolitical units. We investigate a two decade effort to control an outbreak of Chronic Wasting Disease in white-tailed deer populations originating along a geopolitical border. Our work illustrates unexpected outcomes that can arise when differences in management across interconnected social–ecological systems are extreme, which supports continued calls for cooperation across interconnected social–ecological systems through all stages of disease management.

## Introduction

Following recognized importance of wildlife health surveillance, evaluation of disease management remains one of the most pressing and complex challenges of wildlife management (1–5). Diseases in wildlife and human populations are important drivers of social–ecological systems (SESs) (6, 7). Users (i.e. humans) within a SES dictate risk perception, impact tolerance, social acceptability, and cultural and recreational implications of disease management actions (1, 8). Governance systems (i.e. organizations that oversee management actions) can lack the ability to control decisions in adjacent, possibly connected SESs even when there is an inextricable relationship due to mobility of resource units (e.g. wildlife) (9). Both human dimensions and relationships between governance systems complicate how management actions are implemented and to what extent they can be evaluated (1, 2 , 7, 8).

A perhaps under-emphasized challenge in disease management is the comparison of management actions across geopolitical boundaries for the same outbreak (2, 10). In many cases, disease vectors (e.g. birds, insects, and animals) can freely cross geopolitical boundaries, and superficially, it is reasonable to suspect that lack of cooperation may hinder the effectiveness of any management. In many instances, evaluation of disease management requires extensive surveillance over large regions and long periods of time (1, 11). Spatial and time constraints of surveillance efforts are further challenged by other attributes of a disease such as transmission rate, rate of fatality, incubation time, and magnitude of wildlife interaction (4).

In a hypothetical effort to illustrate importance of cooperative disease management across interconnected SESs, we could consider a designed experiment. In this experiment, we can randomly assign disease management strategies to each SES, introduce an outbreak at a shared border of both regions, and enforce standardized surveillance practices. However, there are a number of challenges to implement such an experiment, and there is an opportunity to consider similar observational data where a natural experiment occurred. By natural experiments, we refer to studies in which treatment assignment is determined by events that researchers cannot control (e.g. natural events or policy changes), and there is potential to explore cause–effect relationships (12). An exemplary natural experiment in wildlife disease management might have the following: (i) an outbreak of a slow-moving disease that can be monitored sufficiently, both spatially and temporally, in adjacent geopolitical units, (ii) an outbreak that emerges near or at a shared boundary, and (iii) substantial differences in disease management practices in both geopolitical units. We would consider any study with these criteria to be an exemplary natural experiment in disease management.

**Fig. 1.**
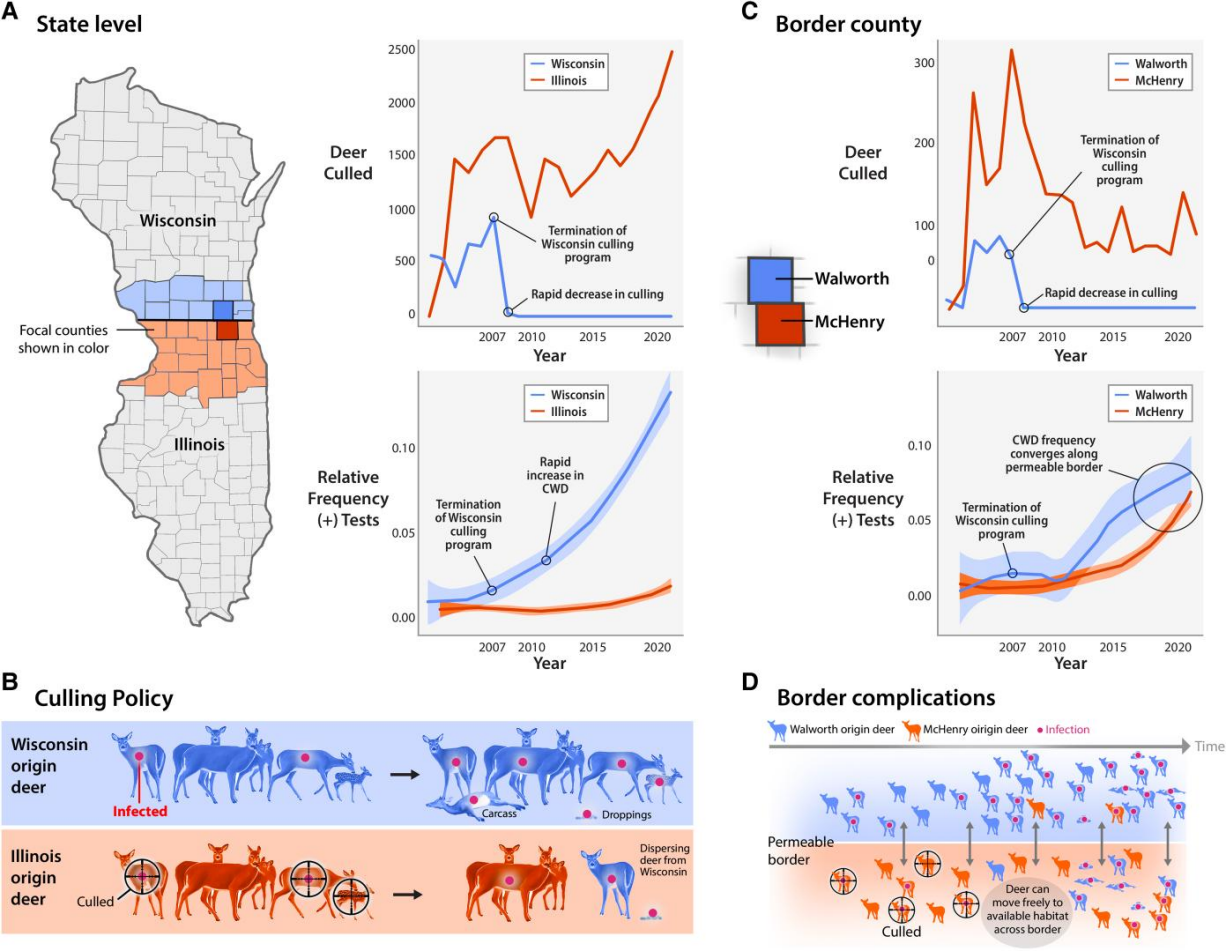
An outbreak of CWD in white-tailed deer was detected near the Illinois–Wisconsin border in 2002. The suspected region of the outbreak was within kilometers of the border near McHenry County, IL and Walworth County, WI (A). Targeted culling efforts (B) aimed at reducing population density and removing infected individuals increased in Illinois over two decades while Wisconsin almost exclusively relied on public hunting after 2007. Following the steep decline in Wisconsin’s culling and total harvest (i.e. deer harvested via public hunting) in 2007, a steep increase in the relative frequency of positive cases occurred, which was not experienced in Illinois (A). However, this result does not hold for some adjacent border counties near the outbreak (e.g. McHenry, IL and Walworth, WI in C), which fail to show the same disparity across a permeable geopolitical border (D). Shaded regions in the lower plots in (A) and (C) represent confidence intervals for expected relative frequency of positive tests using local polynomial regression.

From 2002 to 2022, one such natural experiment occurred on the Illinois–Wisconsin border. Chronic wasting disease (CWD) was detected in both Illinois and Wisconsin in 2002 within kilometers of the shared border in McHenry, IL and Walworth, WI (Fig. 1A). By 2003, there were 14 detected cases across three border counties in Illinois (i.e. Boone, McHenry, Winnebago) and 11 detected cases across four border counties in Wisconsin (i.e. Green, Kenosha, Rock, Walworth). Both states responded with similar disease management actions, including hunting regulations intended to increase hunter harvest and targeted culling (i.e. removal of potentially infected deer by sharpshooting as illustrated in Fig. 1B) (13). In 2007, public resistance and declining legislative support caused the Wisconsin Department of Natural Resources to terminate its culling program and curtail hunter-harvest (Fig. 1A). Following the termination of Wisconsin’s culling program, hunter-harvest regulations led to a 30.1% decline in total harvest from 2007 to 2011 (Fig. S1). The term total harvest refers to the number of deer harvested via public hunting. During the past 15 years, management actions in Wisconsin have shifted to primarily public hunting (14–16).

In this natural experiment, Illinois has continued to use targeted culling and public hunting to control CWD (17). Illinois’ total harvest declined 13.6% from 2007 to 2011 (Fig. S1), but counties along the border experienced a relatively stable harvest (Fig. S2). Over the course of two decades, Illinois and Wisconsin have collectively tested over 220,000 deer for CWD in counties that have cases most likely connected to the 2002 state-line outbreak.

In Wisconsin, the sharp change in management in 2007, illustrated in Fig. 1A, was promptly followed by steep increases in the relative frequency of positive cases in affected counties. Despite continued culling and more stable public harvest in Illinois (Fig. S1), the prevalence of CWD across affected areas slowly increased over time. In juxtaposition, examination of two adjacent Illinois–Wisconsin counties (e.g. McHenry, IL and Walworth, WI) reveals no stark differences in the relative frequency of positive cases at the border (Fig. 1C). At the county-level, declines in targeted culling are reported in McHenry County after 2007 as relative frequency of cases increased in other counties across Illinois resulting in the agency shifting resources to these areas (Fig. S3).

Without statistical analysis at the state-level, we meet many of the criteria to describe a potentially causal relationship between management actions (i.e. total harvest and culling effort) and the relative frequency of positive cases (18, 19). (i) There is a potentially strong relationship between declines in total harvest and culling and positive cases (Fig. 1A and B). (ii) There are no other known events in 2007 that could lead to such disparity in future positive cases for the same outbreak. (iii) Termination of targeted culling and decline in total harvest in Wisconsin occurred before the rapid increase in positive cases. (iv) With minimal natural predation in this region, it is plausible that the combination of higher total harvest and targeted culling could sufficiently lower population density and the number of infected individuals in a region (20, 21). (v) The outlined cause-effect relationship between targeted culling and positive cases does not necessarily contradict the biology of the disease. Further, the later increases in relative frequency of positive cases in Illinois could be attributed to increasing environmental contamination, which total harvest and targeted culling efforts cannot directly mitigate (22).

In spite of this potential claim of causality, it evidently has lesser, if any, applicability on finer scales (e.g. county-level). As shown in Fig. 1C, management decisions appear to have confounding outcomes near the border, where potentially abrupt shifts in management exist across a border with no natural or artificial barriers (Fig. 1D). The stark change of inference as each state is stratified into counties illustrates an exceptional example of the modifiable areal unit problem (MAUP) in spatial data analysis (23). The disparity between state and county-level inference could potentially be explained by a “border effect” resulting from extreme differences in management actions across a state-line. Understanding this inversion of inference further for border counties has the potential to illustrate the importance of cooperative disease management across SESs and implications of failing to do so. A model-based approach has the potential to uncover relationships between management actions and outcomes over time and how these relationships might vary spatially.

Total annual harvest of deer from public hunting and culling is the primary means by which state agencies manage herd size and health within each county. Our findings illustrate an unprecedented investigation into a border effect phenomena arising from the relationship between disjoint management actions and cumulative incidence of a wildlife disease (Fig. 1D). In general, it is hypothesized that increases in total harvest in a county will reduce cumulative incidence of CWD. We investigate this hypothesis using a machine learning-based epidemiological cumulative incidence model. We quantify the importance of the predictive relationship between management actions of Illinois and Wisconsin and the probability that an individual deer has a CWD-positive tissue sample over their potential lifespan (i.e. cumulative incidence). We support our claims of causality using this model, and we illustrate changes in effectiveness of management from 2002 to 2022 at the county-level. With two decades of data documenting the increasing rate of positive CWD tests in Illinois and Wisconsin, we believe we are well-positioned to gain insight about unique challenges of disease management across geopolitical units.

## Results

**Fig. 2.**
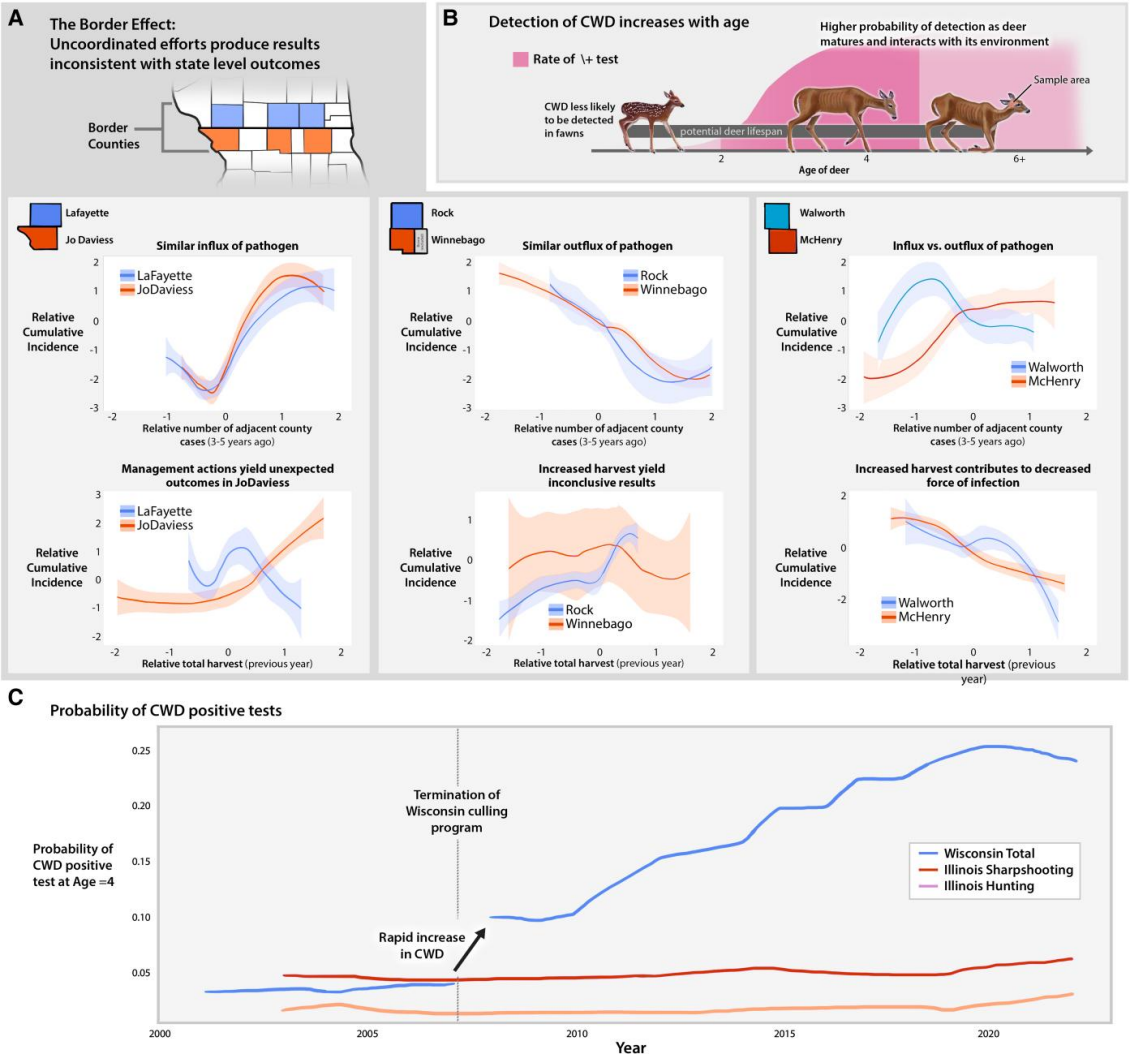
County-level results from our model provided evidence of a spatially heterogeneous border effect. The relationship between total harvest and cumulative incidence illustrated an example of the modifiable areal unit problem, where inference can differ at the state and county-level as well as across counties (A). From the state agency perspective, harvesting of deer via public hunting or culling efforts was aimed at removing infected deer (B) and reducing population density. In some cases, these efforts may induce sink-source dynamics where increases in harvest yield increases in cumulative incidence (e.g. JoDaviess, IL). At the state-level, our model detected a change point in cumulative incidence in Wisconsin following termination of their culling program (C). This detected border effect phenomena illustrates the importance of cooperation of management across geopolitical to support mutually beneficial actions.

Increasing deer harvest through public hunting or culling is considered a viable option for preventing and managing CWD (24, 25). Decreasing deer population density and removal of infected deer early in their clinical course of the disease are often important objectives (Fig. 2B). However, we found that the effects of increasing total harvest at the county-level were highly heterogeneous for counties along the border (Fig. 2A). Border counties did not consistently have a positive relationship between total harvest and cumulative incidence in following years.

JoDaviess, IL and LaFayette, WI are one intriguing pair of adjacent counties along the border. The number of positive cases detected in adjacent counties in recent years predicted virtually identical steep increases in cumulative incidence of CWD (Fig. 2A). This is likely attributed to a known westward spread of CWD to this region, which originally had no detected cases. However, relationships between total harvest and cumulative incidence were opposite for these adjacent counties. JoDaviess is a county that retained a relatively high total harvest and experienced steep increases in culling during the study period as CWD positive deer were detected. Although both counties experienced steep increases in cumulative incidence of CWD over time, our model indicated that increases in total harvest appeared to help control CWD in LaFayette, while in JoDaviess such increases showed evidence of producing the opposite of intended outcomes. This phenomenon was not consistently apparent in other pairs of border counties (Fig. 2A).

McHenry, IL and Walworth, WI counties provided an exceptional illustration of virtually the opposite phenomena exhibited by JoDaviess, IL and LaFayette, WI. The number of positive cases detected in adjacent counties in recent years predicted virtually opposite trends in cumulative incidence of CWD (Fig. 2A). As the number of cases increased in adjacent counties to McHenry, IL, cumulative incidence was predicted to increase, while in Walworth, WI, increasing positive cases in adjacent counties predicted decreases in cumulative incidence. McHenry is only bordered by two other counties that experienced comparable rates of CWD positive tests (Rock County, WI and Boone County, IL) in the past 20 years. Therefore, increasing positive cases in Walworth and Boone County were strong predictors of increased cumulative incidence in McHenry County in subsequent years. We believe this provides evidence of an equilibration of cumulative incidence between these counties owing to sink-source dynamics. However, unlike JoDaviess, IL and LaFayette, WI, increasing total harvest in McHenry, IL and Walworth, WI predicted comparable declines in cumulative incidence in following years.

As we attempted to reconcile striking differences in outcomes based on management along a permeable geopolitical border, our findings indicated that county proximity along the border improved the characterization of this phenomenon. Eastern and central counties (i.e. McHenry and Winnebago, IL and Walworth and Rock, WI) were situated at the suspected origin of the outbreak. From 2002 to 2022, there was an apparent spread of CWD westward and southward along the border to regions experiencing a new emergence of CWD (e.g. JoDaviess, IL and LaFayette, WI). In an examination of changes in cumulative incidence over time, our model identified that all border counties experience comparable increases in cumulative incidence (Fig. S4). However, the timing of such increases indicated that Illinois’ more stable harvest and sustained culling efforts delayed these increases for 3–5 years depending on the proximity of the county to the suspected origin of the outbreak. In further investigation, we found that the relationships detected in JoDaviess, IL were also apparent for nonborder Illinois counties (e.g. LaSalle, IL and Lee, IL) that were also farther from the suspected source of the outbreak (Figs. S5–S7).

At the state-level, we gained a broader picture of the effects of management actions on cumulative incidence. Our model detected a change-point in the cumulative incidence of CWD in Wisconsin. This change-point occurred between 2007 and 2008 (Fig. 2C). Both the year of collection and the state where collection occurred were important predictors of cumulative incidence (Fig. S8). The abrupt shift in predicted cumulative incidence, illustrated in Fig. 2C, provided further support for our claim that termination of Wisconsin’s targeted culling program and declines in total harvest potentially caused abrupt and enduring increases in cumulative incidence.

## Discussion

### Gathering lessons from 20 years of CWD management

Illinois and Wisconsin have both faced significant and unique challenges in managing the spread of CWD. Near the beginning of the CWD state-line outbreak, Wisconsin had already been documenting and managing the spread of a larger CWD outbreak in Central Wisconsin for 2 years (26, 27). Wisconsin faced substantial public resistance and political headwinds, which forced Wisconsin’s Department of Natural Resources to re-evaluate how to manage CWD in the early stages of the borderline outbreak (28 , 29). Illinois had capacity to manage with lesser public and political resistance for almost 20 years, but their more aggressive management strategy challenged available management resources. By management resources, we refer to funding, personnel, equipment, and facilities required for removal, testing, and processing of deer. As the probability of CWD-positive tests in Wisconsin rapidly increased as a result of both the state-line and central Wisconsin outbreaks, Illinois chose to continue with the same management strategy even though Wisconsin lacked the ability to continue with such an approach.

There are few opportunities to reflect on the challenges of such unique, “real-world” (i.e. nonhypothetical) disease management dilemmas. To the best of our knowledge, there are few slow-moving wildlife disease outbreaks as rigorously documented as CWD. In simplest terms, we show that lack of cooperation has potential to yield highly heterogeneous management outcomes. In some cases, management outcomes could have the opposite of the intended effect. We do not suggest that either state’s independent choice to manage more passively or aggressively was the best choice (30). Instead, we believe that this “tale of two management programs” enhances an important discussion in disease management philosophy. Both state’s efforts have provided an overdue opportunity to further discuss important epidemiological and ethical questions of disease management:

1. Can the level of interconnectivity between SESs be determined, and how can governance systems in interconnected SESs use such information to determine need for cooperation?
2. If a geopolitical unit has the resources and public support to manage a disease aggressively, should they do so if a neighboring geopolitical unit lacks the ability to also manage aggressively?
3. For disease outbreaks in which carriers can easily cross artificial borders, what responsibility do both geopolitical units have in cooperatively planning disease management?
4. How can adjacent geopolitical units determine mutually beneficial management actions with potential disparities in public and political support?

Although we can only hypothesize the value of increasing cooperative management, two decades of CWD management in Illinois and Wisconsin illustrate an example of what outcomes we can expect if cooperation across geopolitical units cannot be improved (30, 31). Wisconsin’s abrupt termination of their targeted culling program and aggressive hunter-harvest policies was followed by an increase in cumulative incidence in the Southern part of the state. Illinois’s efforts to continue targeted culling show evidence of yielding a persistent slower increase to cumulative incidence. Perhaps, most importantly, it is evident that lack of cooperation has potential to produce highly heterogeneous management outcomes. In some cases, the outcomes of management could have the opposite of the intended effect.

We have identified that the number of cases in adjacent counties in the past 3–5 years and total harvest are both important predictors of cumulative incidence in a given county. We believe that these differences in the progression of the borderline CWD outbreak are important and related to the movement of deer in the region (32–34). The movements of dispersing deer and the genetic movement (i.e. gene flow) of deer in the region over multiple generations are both possibly related to the spread of CWD in the region (32, 33, 35–37). Growing literature on the effects of deer removal on population dynamics also provides evidence that removal may yield increases in movement to areas where density of a species is the lowest (38–40).

Many criteria are met to infer a causal relationship between management actions in LaFayette county and the outcomes of management actions in JoDaviess county. (i) As identified in our model, there was high predictive importance between the number of positive tests in adjacent counties, total harvest in the previous year, and cumulative incidence. (ii) Other than substantial differences in total harvest and culling efforts, there are few noteworthy geographic and environmental differences between these counties (41). (iii) The number of positive tests 3–5 years ago in adjacent counties and total harvest in the previous year clearly precede the collection and testing of deer in a county. (iv) It is a plausible hypothesis that increasing harvest and culling efforts in JoDaviess County could sufficiently lower population density of deer and catalyze a population sink inducing movement of dispersing deer in adjacent counties with higher deer density (e.g. LaFayette County) over multiple generations. Otherwise, there is the possibility that increases in harvest and culling underestimated population growth and were insufficient to reduce cumulative incidence. (v) The outlined cause–effect relationship does not contradict the biology of the disease. Further, if targeted culling in JoDaviess County is catalyzing localized population sinks, then it is plausible that deer dispersing in adjacent areas may move into the region to take advantage of available habitat.

In addition to this discussion, our model was not able to detect a strong relationship between targeted culling effort in previous years and cumulative incidence of CWD for Illinois counties. Variables describing total harvest in previous years had substantially higher predictive importance than variables describing culling. Culling effort likely has a localized effect on cumulative incidence, but such efforts appear to have lesser influence on a county and state-wide scale. The removal of deer via public hunting (i.e. total harvest) was on the order of thousands at the county-level and tens of thousands at the state-level (Figs. S1 and S2). Removal of deer via culling is a small fraction of total removal of deer from the landscape (Fig. 1). Targeted culling placed greater emphasis on removing infected deer from focal areas on the landscape to affect the growth and spread of CWD locally. As the spatial footprint of an outbreak increases, management through culling becomes increasingly difficult due to strain on requisite resources.

Illinois’ efforts to sustain harvest levels and aggressively cull (i.e. to lower population density) and Wisconsin’s rapid termination of their targeted culling program may both be jointly responsible for movements that are conducive to increasing prevalence of CWD. We have shown Wisconsin’s abrupt termination of their targeted culling program was the strongest predictor of increasing cumulative incidence. Prediction of cumulative incidence in a county ultimately requires that we look to the events occurring in neighboring counties on both sides of the border.

We believe that cooperative management efforts of a state-line outbreak may have produced more optimal outcomes for both states. The progression of a CWD outbreak and management decisions of a geopolitical unit are related to the outcomes of neighboring units in the following years. As such, consideration of management actions outside their jurisdiction may benefit cost-risk assessments of management within a county or state (42–44).

In spite of this, we must acknowledge that such lack of cooperation has created an exemplary experiment in disease management. In such scenarios, random assignment of a treatment (i.e. a disease management action) is unlikely. In situations where cooperative management across state agencies increases, there is an inherent increase in dependence of both disease management actions and outcomes. Increasing dependence may reduce our ability to evaluate outcomes of various management actions. This is because cooperating state agencies may coordinate more homogeneous efforts with neither entity being willing to be assigned to a control group. The circumstances surrounding management of the Illinois–Wisconsin outbreak yielded a scenario where strong independence of management actions is evident and differences in management actions were stark. To the best of our knowledge, it is unclear if such a scenario can be manufactured when cooperative management occurs.

## Conclusion: sustainability of disease management—across borders and over time

We believe that information obtained from two decades of CWD management on the Illinois–Wisconsin state-line can help guide the future of disease management to a more productive environment. Consideration and rigorous evaluation of cooperative management decisions across geopolitical units may mutually benefit disease management efforts. We believe that cooperative management would require sharing of relevant data and continuous communication between all cooperating parties about management restrictions, resources, and capabilities. The management of CWD is a multidecadal effort that requires a commitment to sustainability, adaptability, and communication between public and geopolitical entities. Open evaluation of benefits and consequences of disjoint and cooperative disease management efforts could improve future disease management.

## Methods

### CWD case and management data

An evaluation of Wisconsin and Illinois’ disjoint management efforts requires integration of management data from both states (45). Wisconsin’s CWD data from 2002 to 2021 are used alongside Illinois CWD data from 2003 to 2022. Both datasets provided information about age of deer at death, location and time of collection information, and a categorical variable describing collection type.

Ages of deer were assigned as a discrete quantitative variable, where fawn (i.e. age = 6–8 months) was the youngest age level and older ages were rounded to a 1 year age range. Approximately 38% of deer culled in Illinois were labeled as fawns and 51% were labeled as either fawns or 1-year old. Collectively, in both Illinois and Wisconsin, ∼40.9% of collected deer were identified as fawns or 1-year old. In the Wisconsin CWD data, age was occasionally rounded to a 3 year age range (e.g. age = 4–6 years). The age distribution of collected deer is dependent on population age structure and preferential sampling inherent to each type of collection. In Wisconsin, location at time of collection was predominantly reported at the section-level (i.e. in 1 mi^2^ units) or at the quarter section-level (i.e. 1/2 mi^2^ units). In Illinois, location was reported either at the section or county-level. For Illinois, collection type for each deer is recorded as public hunting, sharpshooting, roadkill, suspect (removal of individuals exhibiting clinical signs consistent with CWD for testing), or other. For all deer collected in Wisconsin, we have labeled them as either “WI: 2001–2007” or “WI: 2008–present.” In Wisconsin, 3,660 deer out of 74,665 were collected by targeted culling, which almost exclusively took place from 2001 to 2007. The abrupt shift in Wisconsin after 2007 contains information about collection type since most deer in Wisconsin were collected via sharpshooting or public hunting from 2002 to 2007, whereas after 2007 most were collected via public hunting.

We focused on county and state-level inference in our analysis. This is a practical choice for data integration, since all collected deer had county-level location information. Management culling decisions and prioritization in Illinois were accomplished at the county-level. As such, county-level inference is possible with regards to estimating cumulative incidence functions for each individual and characterizing their relationship to management efforts among other factors (26).

### Estimating cumulative incidence functions for CWD test status using random survival forests

For all deer collected in both states, approximate age was assigned and CWD test status was provided. Public hunting and targeted culling are the dominant types of collection, and collectively they are used to reduce population density and remove as many infected individuals as possible from highly localized regions. At the individual level, both public hunting and targeted culling terminate disease progression or infection potential for a respective deer (Fig. 2B). Direct contact and environmental contamination are both responsible for transmission of CWD (22), and our objective is to estimate the probability that a deer tests positive for CWD throughout their potential lifespan given that they evade public hunting or targeted culling efforts.

As an example, collected fawns and 1-y-old deer have likely spent less time interacting with other deer and a potentially contaminated environment. If they are infected, they are also less likely to test positive for CWD than mature deer (35). Fawns and 1-y-old deer culled have an unrealized life history that would explicitly have accumulating hazard of infection. As mentioned before, fawns and 1-y-old deer collectively represent a majority of culled deer in Illinois, and we would like to obtain inference about these deer under the assumption that they were able to evade public hunting and targeted culling efforts. Deer collected with a negative CWD test can be thought of as right-censored observations, where date of collection marks the end of the study period for an individual. Beyond this study period, if they had evaded collection, there is a continued hazard of infection. For right-censored data, time-to-event analysis provides a framework for estimating a cumulative incidence function for each deer that illustrates the probability of a positive CWD test across a potentially unrealized life history.

Time-to-event analysis, also referred to as survival analysis, is one option for modeling the relationship between a collection of variables and a censored response variable (46). The observed data are (T,δ), where *T* is the observed event time and *δ* is a censoring indicator variable. The observed event is represented by $T=\text{min}(C_{0},T_{0})$, where $C_{0}$ is the censoring time and $T_{0}$ is the “true, potentially unobserved” time-to-event. When δ=1, the event has occurred and $T=T_{0}$. When δ=0, the event has not occurred and $T=C_{0}$. In our case, the indicator variable *δ* is CWD test status of an individual and observed event time is deer age at time of collection.

In general, a primary objective of a time-to-event analysis is to estimate a survival function for each individual S(t|X). In our work, we refer to 1−S(t|X) as cumulative incidence. Cumulative incidence is the probability that an individual is CWD-positive by some time *t*. This is represented by $1-S(t\;|\;X)=1-P(T_{0}>t\;|\;X)=1-\int_{t}^{\infty }f(t)dt$, where f(t) is the probability density function of the random variable *T* (46). Aggregating estimated cumulative incidence over all individuals tested in a region represents the proportion of a population that is CWD positive over some time period.

To estimate cumulative incidence, we used a random survival forests (RSF) model, which allows us to harness the advantages of an interpretable machine learning approach (47–49). RSFs extend the principles of random forest regression to data with a right-censored time-to-event response, where an ensemble of bootstrapped time-to-event trees are grown using random feature selection. The time-to-event trees are similar to regression trees, the standard base-learner for random forest regression, except the splitting rule must account for right censoring of the data (50, 51). Within each time-to-event tree, individuals are split into groups based on similarity of their time-to-event behavior (50, 52).

Using RSFs allows use of variable importance and partial dependence to characterize the predictive relationship between an array of variables (Table 1) and cumulative incidence for each individual. By incorporating year and an array of spatial variables in the model, the RSF can be thought of as a coarse representation of the spatially and temporally dynamic progression of CWD. Our intent with this model is to derive county and state-level inference of cumulative incidence over time. This is achieved by aggregating estimated cumulative incidence functions for each individual at the county and state-level, respectively. We believe that it is valuable and pressing to determine if hunter harvest and targeted culling efforts yield discernible broader-scale changes in cumulative incidence using widely accessible methodology. Further, we are interested in identifying the importance of hunter harvest and targeted culling efforts in relationship to other variables in the model in estimating the cumulative incidence function in subsequent years.

**Table 1.** Description of variables used in cumulative incidence chronic wasting disease model.

Variable	Definition
Border length	Length of county’s state-line
Collection type	Description of deer death type
County ID	–
Latitude	Latitudinal location of county centroid
Longitude	Longitudinal location of county centroid
State ID	–
Year	Date of collection/deer death
Total harvest: Past year	–
Total harvest: 1–3 years ago	–
Total harvest: 3–5 years ago	–
State prioritization	Percentage of state total culled within a county 1–3 years ago
State prioritization	Percentage of state total culled within a county 1–3 years ago
No. of culled: Past year	–
No. of culled: 1–3 years ago	–
No. of culled: 3–5 years ago	–
No. of of adjacent Cty cases: 1–3 yrs	–
No. of of adjacent Cty cases: 3–5 yrs	–
No. of culled adjacent Cty’s: 1–3 yrs	–
No. of culled adjacent Cty’s: 3–5 yrs	–

### Construction of a spatiotemporal lagged case and management-related covariates

Surveillance of CWD and targeted culling efforts required substantial effort across space and over multiple years (22, 26). Total harvest and targeted culling efforts are intended to reduce population density of deer and control CWD in following years. As such, lagged effects of reported positive cases, total harvest, and targeted culling efforts over both space and time on the probability of CWD-positive tests are of important consideration. In Table 1, we list all temporal and spatio-temporal effects used in our cumulative incidence model. We consider five types of lag effects for a given county: (i) total harvest, (ii) the state prioritization of culling effort, (iii) the culling effort, (iv) the culling effort in all adjacent counties, and (v) the number of cases in all adjacent counties. Some lag effects were constructed to measure each quantity aggregated over two 3-year periods: 1–3 years ago and 3–5 years ago.

As shown in the Supporting Information (Fig. S3), state prioritization of culling effort was subject to change as the probability of CWD-positive tests of CWD increased in counties that were not at the location of the cases reported in the initial outbreak. For Illinois and Wisconsin, we have the number of deer culled per county for all counties depicted in Fig. 1A. We constructed lagged state culling prioritization variables by summing all deer culled in each county for 3-year periods, and then we computed the percentage of deer culled in each county. All remaining lagged effects are simple sums of the number of deer culled or the number of positive cases aggregated over each time period.

We consider the listed lagged effects for the following reasons: (i) they are all easily computed from data immediately available to wildlife managers, (ii) they provided best possible description of county-level culling efforts and the state of CWD in surrounding counties that Illinois and Wisconsin’s Department of Natural Resources could document each year, (iii) they are easily interpreted. Collectively, (i–iii) are important as we have a pertinent interest in which lag effects have the highest relative importance at predicting when deer in a given county test positive for CWD over their potential lifespan.

## Supplementary Material

pgaf387_Supplementary_Data1

pgaf387_Supplementary_Data2

pgaf387_Supplementary_Data3

## Data Availability

The Wisconsin Department of Natural Resources and Illinois Department of Natural Resources independently clean, curate and maintain their individual databases. Data supporting the Wisconsin findings of this study are available from Daniel Storm (danielj.storm@wisconsin.gov) at the Wisconsin Department of Natural Resources. Data supporting the Illinois findings of this study are available from Chris Jacques (chris.Jacques@illinois.gov) at the Illinois Department of Natural Resources. Chronic wasting disease hunter-harvest data contains sensitive information about presence of disease in areas that span private and public areas. Release of data by Wisconsin DNR and Illinois DNR is done on a case by case basis. We have included harvest data for both states with our submission, which is publicly available.
