# Supplementary material for: A tale of two management programs: Insights from a state-line wildlife disease outbreak
Source: PNAS Nexus. 2025 Dec 10;4(12):pgaf387. doi: 10.1093/pnasnexus/pgaf387 (PMC12727682; doi:10.1093/pnasnexus/pgaf387)
Supplement: pgaf387_Supplementary_Data1 [file pgaf387_supplementary_data1.pdf]

Supporting Information for

A tale of two management programs: Insights from a state-line wildlife disease outbreak

Andrew B. Whetten, Trevor J. Hefley, Christopher N. Jacques, Daniel J. Storm, Daniel P. Walsh

Corresponding Author:
Email: abw225@cornell.edu

This PDF file includes:

Supporting text
Figures S1 to S8
Legends for Datasets S1 to S5
SI References

Other supporting materials for this manuscript include the following:

Datasets S1 to S6
Software S1 to S2

Supporting Information Text

We have used a standard survival analysis procedure and the random survival forest algorithm. Our machine learning based survival analysis is related to other literature on force of infection modeling and machine learning based modeling of Chronic Wasting disease data (Heisey et al. 2010, Ahmed et al. 2024). The premise of our survival analysis is that detection of CWD in an individual deer is a potentially right-censored observation. Within this approach, the date of testing is treated as an artificial end to the monitoring of each deer that has a potential lifespan and some probability of testing positive for CWD at any time. We encourage readers to refer to the following references for survival analysis and random survival forests (Landes et al. 2024, Ishwaran et al. 2008, Ishwaran andd Kogalur 2024)

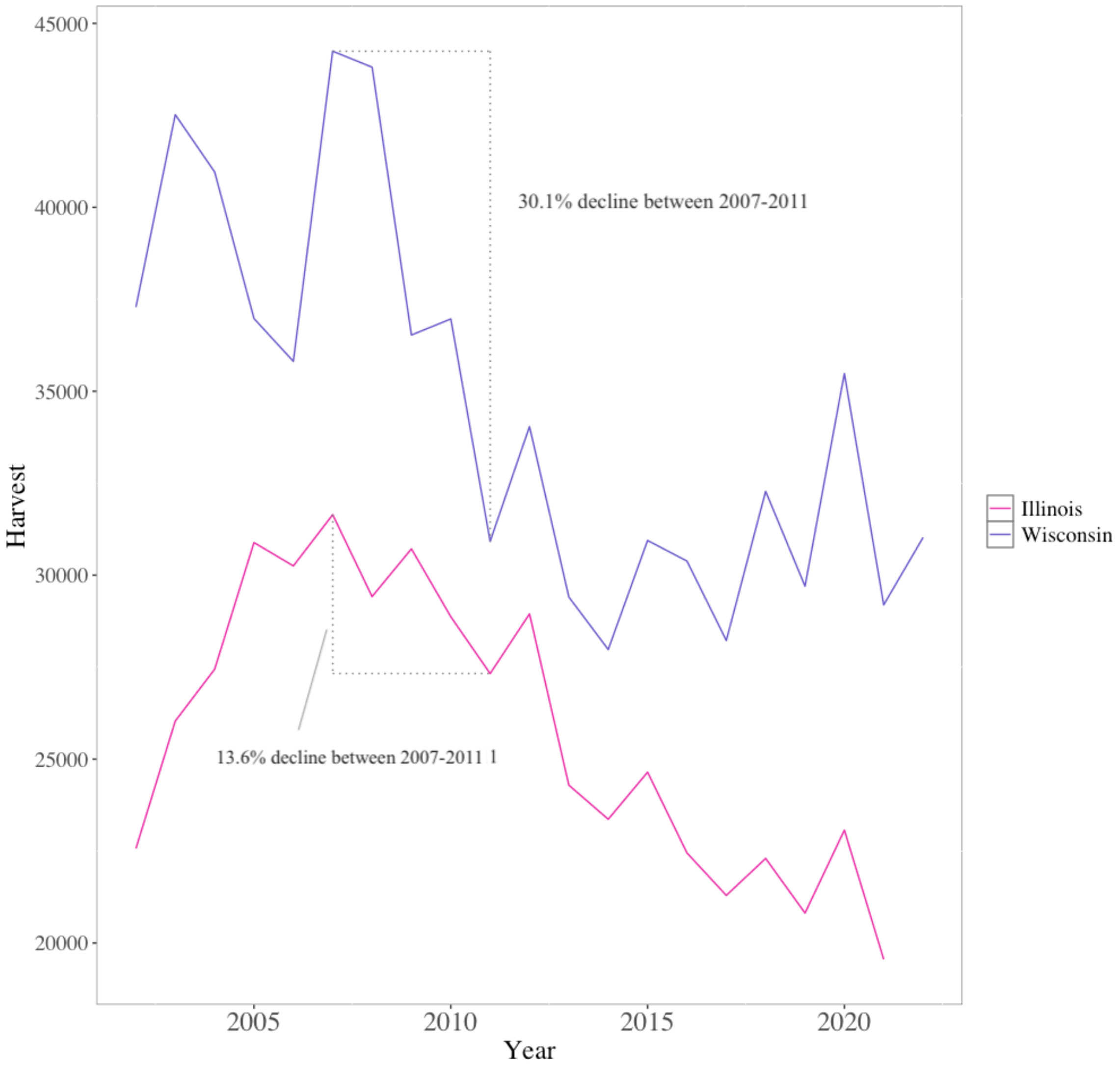

Fig. S1. State Level Harvest 2003-2021. Following the termination of Wisconsin's culling program in 2007, culling abruptly ended within the next year (Figure 1A). The termination of Wisconsin's culling program was also followed by a steep decline in total harvested deer by public hunting. Wisconsin's total harvest declined by 30.1% between 2007-2011. Illinois' total harvest decreased by 13.6% during the same time period, but culling totals were sustained and steadily increased between 2010-2021 (Figure 1A). The termination of the culling program and the steep decline in total harvest in Wisconsin mark a major shift in the management efforts between 2007-2011. Counties used to compute the illustrated Wisconsin state-level harvest are as follows: Dane, Grant, Green, Iowa, Jefferson, Kenosha, Lafayette, Racine, Rock, Walworth, and Waukesha. This list comprises of most Southern Wisconsin counties. Counties used to compute the illustrated Illinois state-level harvest are as follows: Boone, Bureau, Carroll, Cook, Dekalb, Dupage, Grundy, Henry, JoDaviess, Grant, Kane, Kendall, Lake, Lasalle, Lee, McHenry, Ogle, Putnam, RockIsland, Stevenson, Whiteside, Will, Winnebago. This list comprises of most Northern Illinois counties.

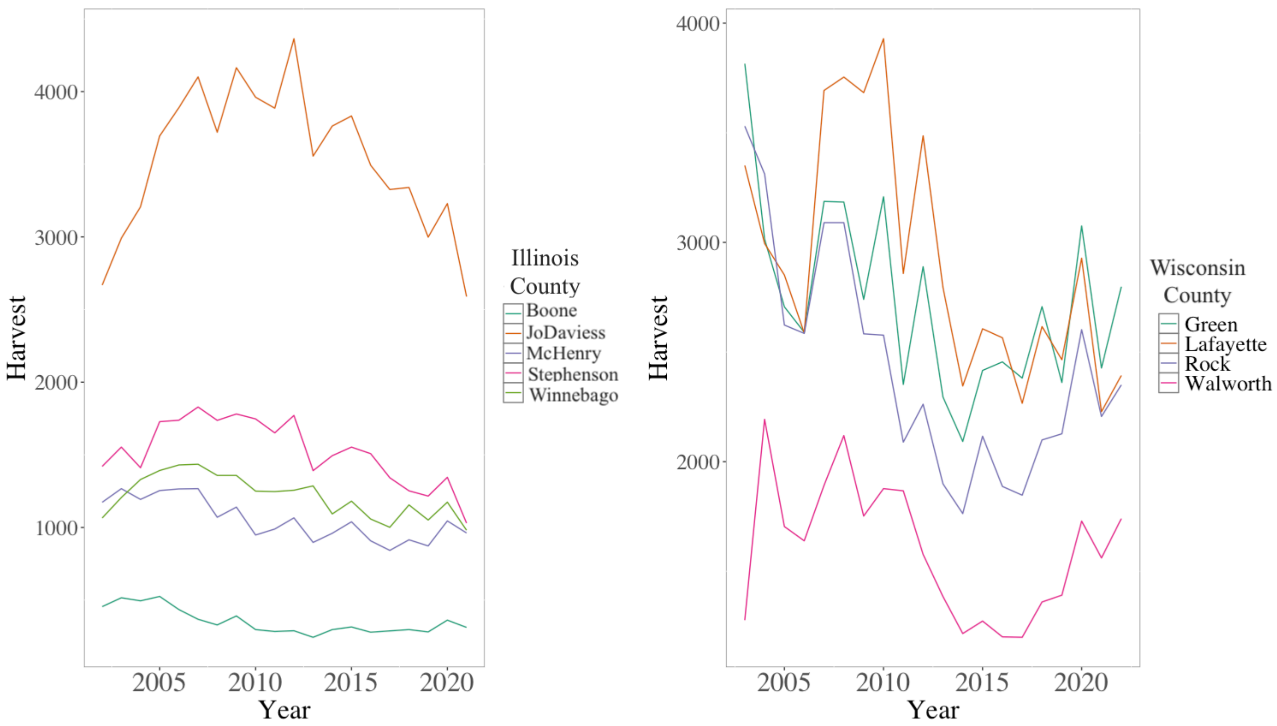

Fig. S2. County Level Harvest for Illinois border counties 2003-2021 generally remained stable. In some cases, total harvest increase between 2007-2012. All shown Illinois counties experienced some long-term decline in total harvest. County Level Harvest for Wisconsin border counties steeply declined sometime between 2008-2011 depending on the county. As an example, steep declines in total harvest took place as early as 2008 for Rock, WI, whereas steep declines in total harvest did not occur until approximately 2011 for Walworth and Lafayette, WI.

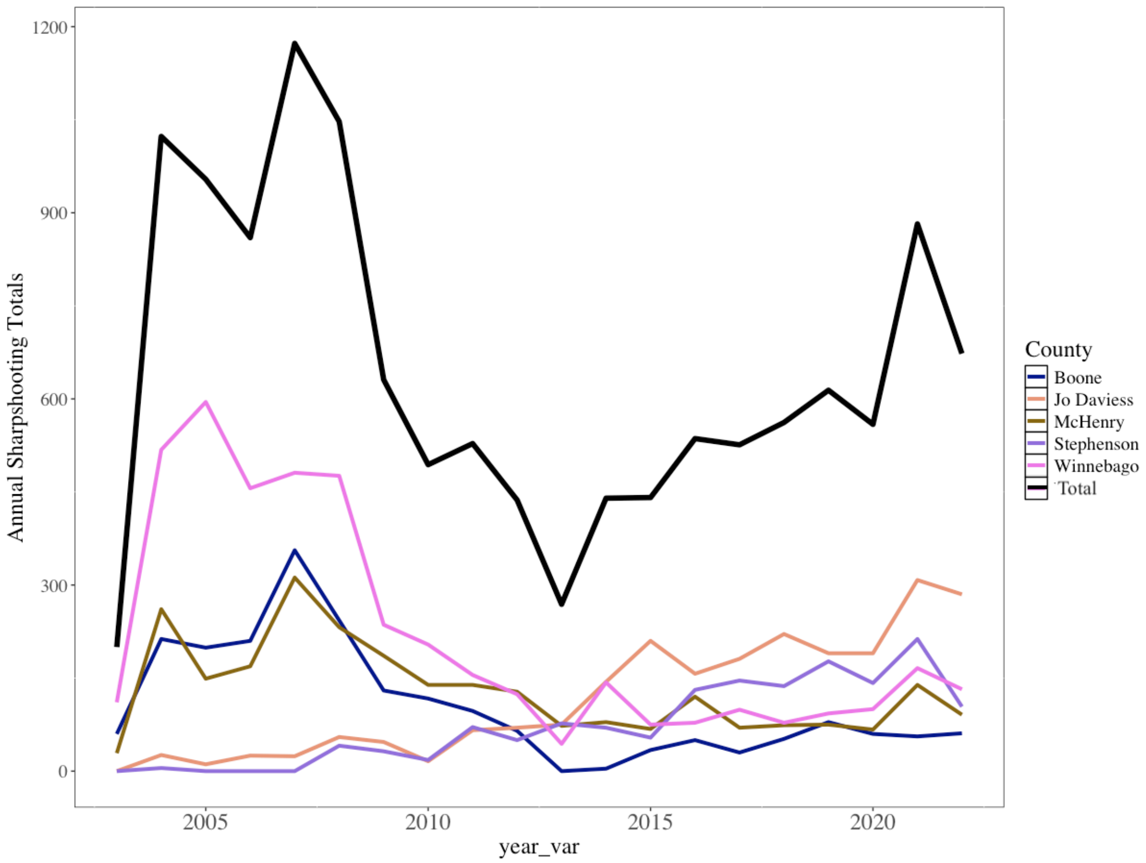

Fig. S3. Illinois' culling efforts along the border were subject to substantial change over time as CWD spread farther westward to JoDaviess, IL. Border county culling reached its peak in 2008 with most culling effort concentrated in counties near the suspected origin of the outbreak in Boone, McHenry, and Winnebago, IL. Border county culling decreased in the middle of the study period. Culling steadily increased between 2014 to 2021 with a substantial proportion of culling occurring in JoDaviess, IL.

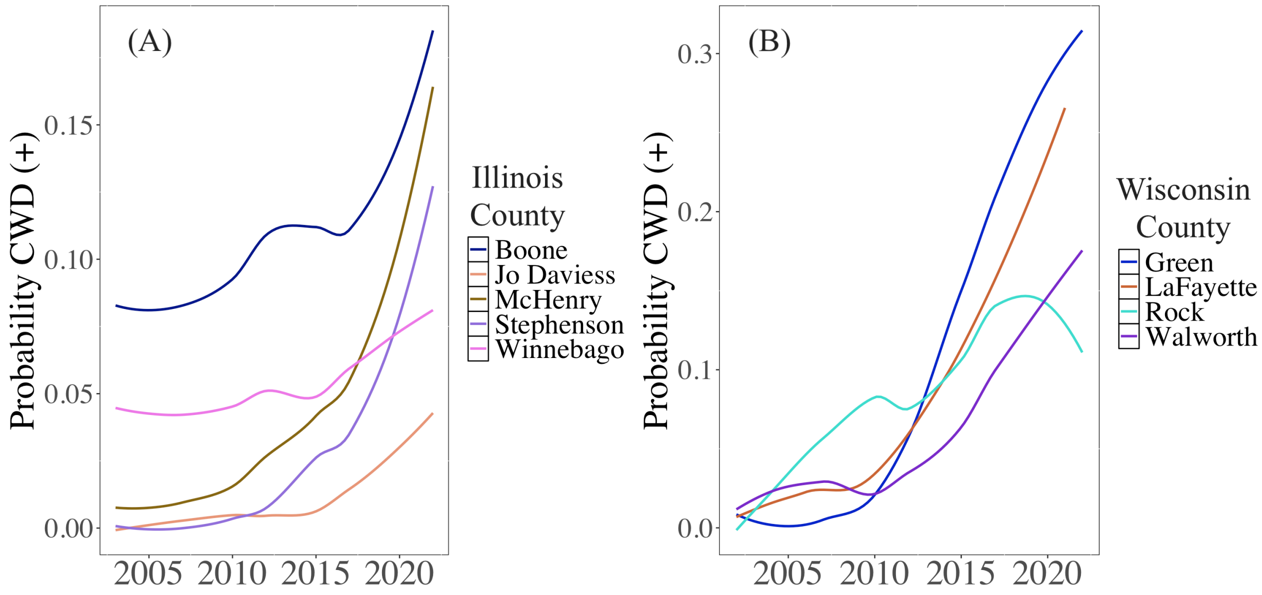

Fig. S4. County level cumulative incidence has increased along both the Illinois and Wisconsin sides of the border. Our model indicates that cumulative incidence for Wisconsin border counties generally began to increase rapidly beginning in 2009. For Illinois counties, the timing of steep increases in cumulative incidence appeared to have greater variation. JoDaviess, IL, the farther west county along the Illinois border, began to experience steep increases in cumulative incidence in 2015, whereas the remaining counties began to experience increasing cumulative incidence around 2010-2012. The panels suggest that Illinois's management efforts may have delayed increasing cumulative incidence, but it is less clear whether such efforts will sustain lower force of infection than Wisconsin going forward.

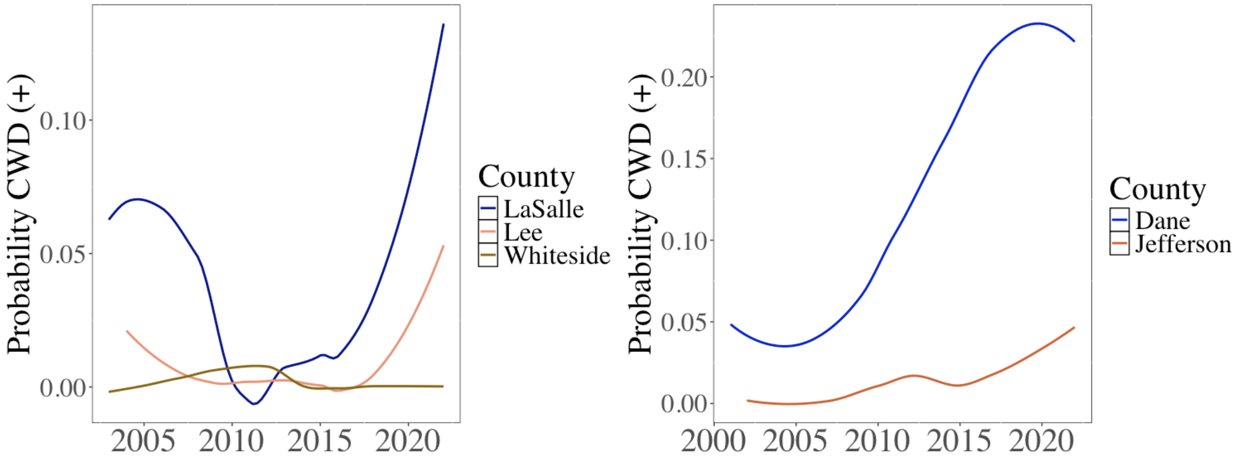

Fig. S5. For non-border counties, cumulative incidence has increased along both the Illinois and Wisconsin sides of the border, but the timing of changes in cumulative incidence is highly disparate relative to adjacent border counties in IL and WI. Steep increases in LaSalle, IL and Lee, IL, are not apparent until 2016-2018, which juxtaposes the steep increases in cumulative incidence along both sides of the border that begin around 2009-2012 as shown in Figure S4. Whiteside, IL is an example of a non-border county that experience less detections of CWD and appears to have maintained lower cumulative incidence across the study period. Dane, WI and Jefferson, WI both have earlier increases in CWD than non-border Illinois counties.

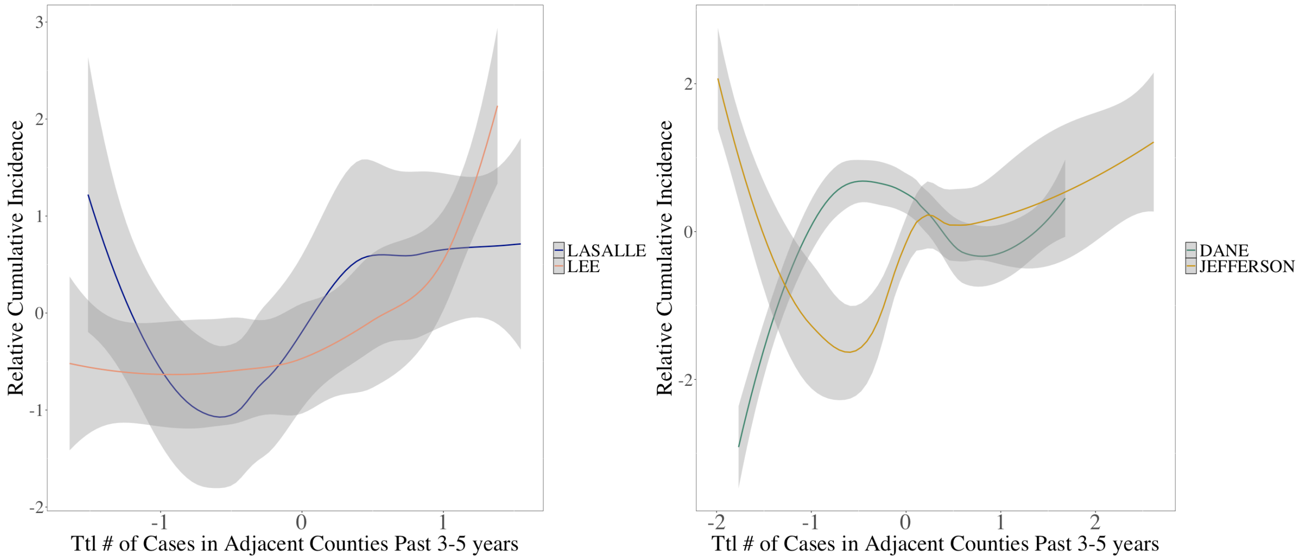

Fig. S6. For non-border IL counties such as LaSalle and Lee, IL, increasing number of cases in adjacent counties is predictive of increases in cumulative incidence. This relationship is also apparent for Jefferson, WI. However, Dane, WI shows evidence of the opposite trend, where increasing number of cases in adjacent counties is predictive of decreasing cumulative incidence.

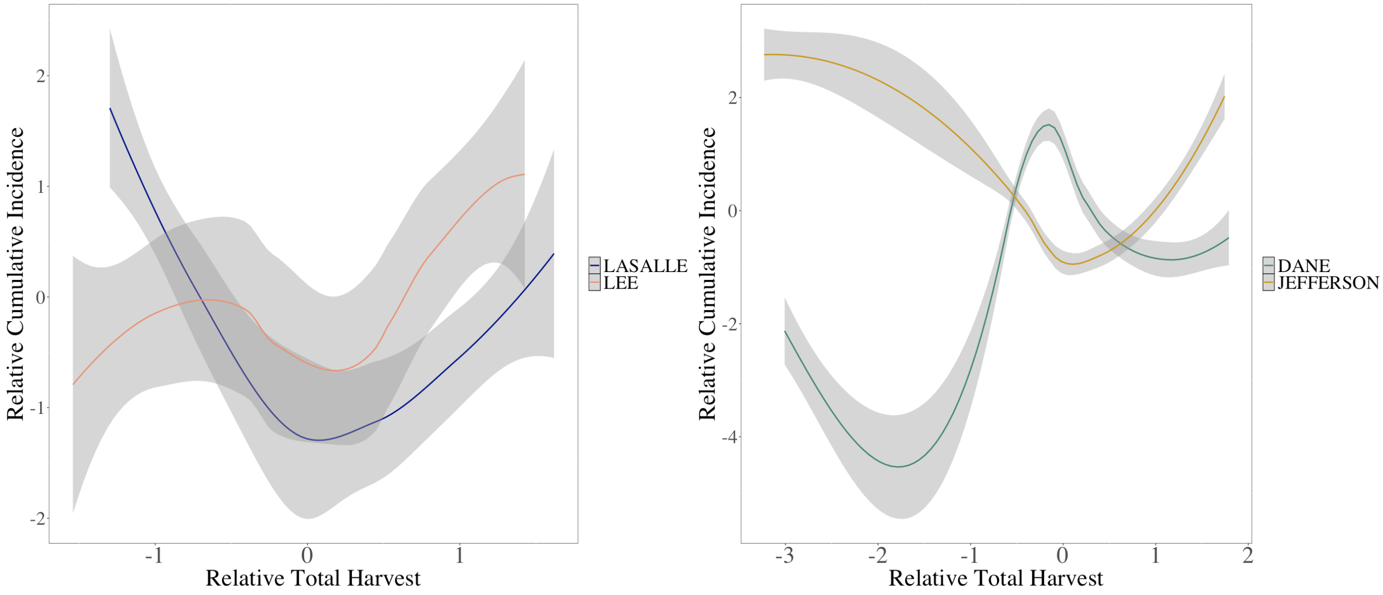

Fig. S7. For non-border IL counties such as LaSalle and Lee, IL, increases to larger than average total annual harvest are predictive of increases in cumulative incidence. This relationship is opposite for Dane, WI where increasing to larger than average harvests are predictive of decreasing cumulative incidence. Jefferson, WI shows a similar relation to that of LaSalle and Lee, IL.

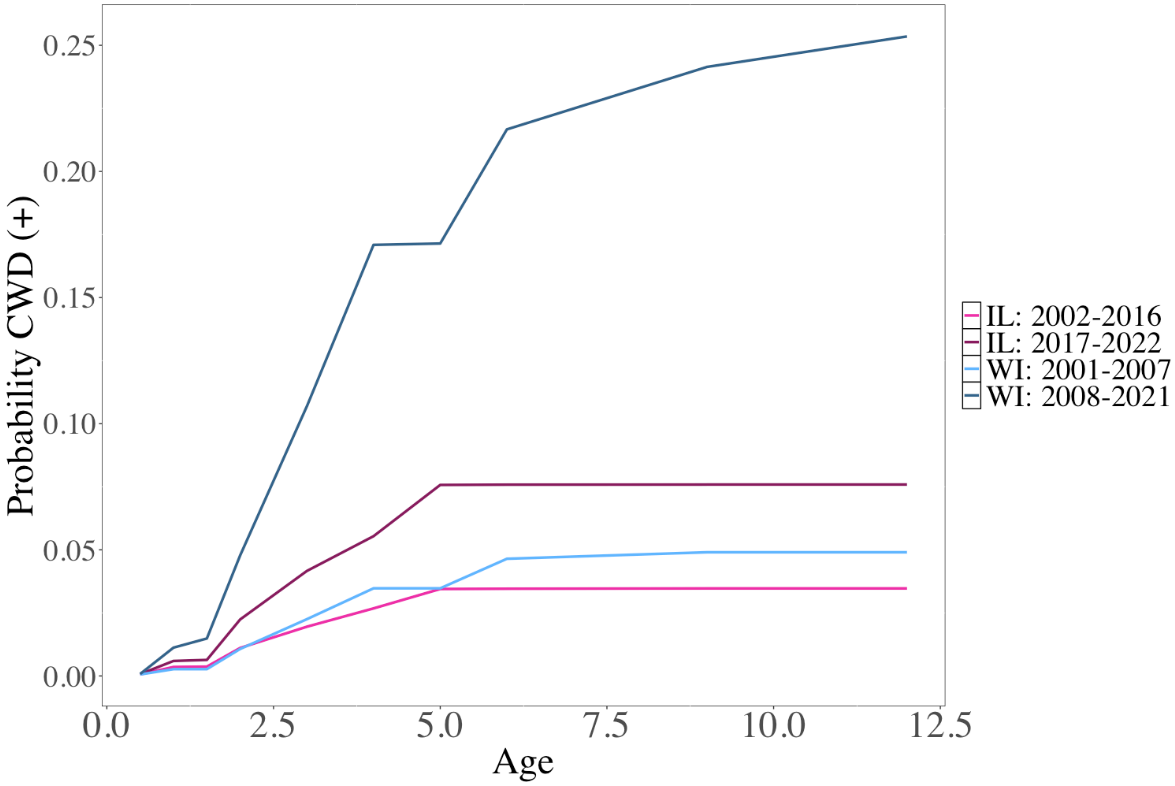

Fig. S8. Our model identified a change point in cumulative incidence at the state-level following 2007 (Fig. 2). For Wisconsin, plotting aggregated inverted survival curves (i.e., $1 - S(t \mid X)$ with respect to the 2001-2007 versus the 2008-2021 periods of time reveals a drastic shift in the probability that individual deer tests positive for CWD during their potential adult life. In Illinois, there is an increase in the probability that individual deer test positive for CWD in later years of data collection, but such increases were delayed and nearly as drastic.

Data Access: The data that supports the Wisconsin findings of this study are available from Daniel Storm (danielj.storm@wisconsin.gov) at Wisconsin Department of Natural Resources. The data that supports the Illinois findings of this study are available from Chris Jacques (chris.Jacques@illinois.gov) at Illinois Department of Natural Resources.

Dataset S1 (access by permission). Illinois Case Data. This dataset organized by the Illinois Department of Natural Resources includes testing information for all deer collected via culling and public hunting during the monitoring period.

Dataset S2 (access by permission). Wisconsin Case Data. This dataset organized by the Wisconsin Department of Natural Resources includes testing information for all deer collected via public hunting during the monitoring period.

Dataset S3 (access by permission). Wisconsin Case Data. This dataset organized by the Wisconsin Department of Natural Resources includes testing information for all deer collected via culling from 2002-2008.

Dataset S4 (separate file). Illinois Harvest Data. Harvest data is published in annual reports. It can be accessed directly from the following link: https://huntillinois.org/deer#deer-management-by-the-numbers**.** Dataset S4, a csv file titled "il_harvest.csv," is the web-scraped and cleaned version of this data.

Dataset S5 (separate file). Wisconsin Harvest Data. This dataset organized by the Wisconsin Department of Natural Resources includes counts of all harvest deer in Wisconsin by county. Dataset S5, a csv file titled "wi_harvest.csv" has been prepared by the Wisconsin Department of Natural Resources for this manuscript.

Software S1 (separate file). This is an R code file that can be used to replicate data cleaning procedure. The file titled "cwd_data_cleaning_explore_visuals.R" documents the cleaning and preparation procedures for the data. This file also include code used to generate components of Figure 1 as well as Figures S1, S2, and S3.

Software S2 (separate file). This is an R code file that can be used to replicate the analysis of the cleaned data. The file titled "cwd_surv_analysis.R" documents the analysis of the data using random survival forests. This file also include code to generate components of Figure 2 and Figures S4 and S5.

SI References

1. MS Ahmed et al, Predicting chronic wasting diseases in white-tailed deer at the county scale using machine learning. Sci. Report 14, 14373 (2024)
2. DM Heisey, et al., Linking process to pattern: Estimating spatiotemporal dynamics of a wildlife epidemic from cross-sectional data. *Ecol. Monogr*. **80**, 221–240 (2010).
3. J Landes, SC, Engelhardt, F Pellender, An introduction to events history analyses for ecologist.
4. H Ishwaran, UB Kogalur, EH Blackstone, MS Lauer, Random Survival Forests. Annals of Applied Statistics. 2, 841—860 (2008).
5. H Ishwaran, UB Kogalur, Fast Unified Random Forests for Survival, Regression and Classification (RF-SRC), (2024) R package version 3.1.0.
