# Supplementary material for: A tale of two management programs: Insights from a state-line wildlife disease outbreak
Source: PNAS Nexus. 2025 Dec 10;4(12):pgaf387. doi: 10.1093/pnasnexus/pgaf387 (PMC12727682; doi:10.1093/pnasnexus/pgaf387)
Supplement: pgaf387_Supplementary_Data2 [file pgaf387_supplementary_data2.pdf]

```
library(geosphere)
library(leaflet)
library(lubridate)
library(spatsurv)
library(survival)
library(sf)
library(sp)
library(gridExtra)


################################################################
################################################################
##### IL CWD Data Processing ###############################
################################################################
# Prepping data for survival analysis
setwd("/Users/andrewwhetten/Desktop/CWD_Case_Data/")
cwd_il <- read.csv("il_cwd_03_22.csv")
head(cwd_il)

str(cwd_il)
table(cwd_il$AGE)

table(cwd_il$HARVESTCO == "COUNTY")
# Age = 1,2,3,4, or 5 we call interval censored where Age = 1 corresponds
# to the interval [1,2)
# Age = A/Adult we call interval censored where Age = A corresonds to
# the interval [1.5, 10]
# Age = U or Unknown, we call interval censored where Age = [1,10]

cwd_il_age_1 <- cwd_il$AGE
cwd_test_event <- cwd_il$TESTSTATUS
cwd_test_event[cwd_test_event=="POSITIVE"] <- 1
cwd_test_event[cwd_test_event=="NOT DETECTED"] <- 0

cwd_il_age_1[cwd_il_age_1=="A"] <- 1.5
cwd_il_age_1[cwd_il_age_1=="U"] <- 0.5
cwd_il_age_1[cwd_il_age_1=="F"] <- 0.5

# Construct Right-Censored Matrix
cen_mat <- cbind(as.numeric(cwd_il_age_1), as.numeric(cwd_test_event))
head(cen_mat, 25)


################################################################
################################################################
##### WI CWD Data Processing ###############################
################################################################
# Prepping data for survival analysis

cwd_wi <- read.csv("wi_cwd_01_21.csv")
head(cwd_wi)

# Data for 2002-2007 in separate csv
```

```
cwd_wi_add_on <- read.csv("culled_cwd.csv")
head(cwd_wi_add_on)

str(cwd_wi)
library(lubridate)
cwd_wi$kill.date_dec <- decimal_date(as.POSIXct(cwd_wi$kill.date, format = "%m/%d/%Y",
tz = "UTC"))
cwd_wi$year <- floor(cwd_wi$kill.date_dec + 2000)
rem1 <- which(cwd_wi$kill.date_dec >23)
cwd_wi <- cwd_wi[-rem1,]

table(is.na(cwd_wi$kill.date_dec))
rem2 <- which(cwd_wi$kill.date == "")
cwd_wi <- cwd_wi[-rem2,]

# repeat for the add_on 2002-2007 WI data
cwd_wi_add_on$Kill.Date<- decimal_date(as.POSIXct(cwd_wi_add_on$Kill.Date, format =
"%m/%d/%Y", tz = "UTC"))
cwd_wi_add_on$year <- floor(cwd_wi_add_on$Kill.Date)
names(cwd_wi_add_on)
names(cwd_wi)

table(is.na(cwd_wi_add_on$Kill.Date))
rem3 <- which(is.na(cwd_wi_add_on$Kill.Date) == TRUE)
cwd_wi_add_on <- cwd_wi_add_on[-rem3,]

# Age = 1,2,3,4, or 5 we call interval censored where Age = 1 corresponds
# to the interval [1,2)
# Age = A/Adult we call interval censored where Age = A corresonds to
# the interval [3.5, 10]
# Age = U or Unknown, we call interval censored where Age = [1,10]
table(cwd_wi$age)
table(cwd_wi$positive)
cwd_wi_age_1 <- cwd_wi$age

cwd_test_event <- cwd_wi$positive
cwd_test_event[cwd_test_event=="Y"] <- 1
cwd_test_event[cwd_test_event=="N"] <- 0

cwd_wi_age_1[cwd_wi_age_1=="5-Apr"] <- 4

cwd_wi_age_1[cwd_wi_age_1=="8-Jun"] <- 6

cwd_wi_age_1[cwd_wi_age_1=="11-Sep"] <- 9

cwd_wi_age_1[cwd_wi_age_1=="12+"] <- 12

cwd_wi_age_1[cwd_wi_age_1=="ADULT"] <- 1.5

cwd_wi_age_1[cwd_wi_age_1==""] <- 0.5

cwd_wi_age_1[cwd_wi_age_1=="F"] <- 0.5

#################
```

```
### Add-on data ##
# For Wisconsin 2002-2007 add-on data
table(cwd_wi_add_on$Age)
table(cwd_wi_add_on$Result)
cwd_wi_age_02 <- cwd_wi_add_on$Age

cwd_test_event02 <- cwd_wi_add_on$Result
cwd_test_event02[cwd_test_event02=="Inconclusive Result"] <- 0
cwd_test_event02[cwd_test_event02=="Negative"] <- 0
cwd_test_event02[cwd_test_event02=="No Result"] <- 0
cwd_test_event02[cwd_test_event02=="No Samples Collected"] <- 0
cwd_test_event02[cwd_test_event02=="Not Detected"] <- 0
cwd_test_event02[cwd_test_event02=="Positive"] <- 1

cwd_wi_age_02[cwd_wi_age_02=="4 to 5"] <- 4

cwd_wi_age_02[cwd_wi_age_02=="6 to 8"] <- 6

cwd_wi_age_02[cwd_wi_age_02=="9 to 11"] <- 9

cwd_wi_age_02[cwd_wi_age_02=="12+"] <- 12

cwd_wi_age_02[cwd_wi_age_02=="ADULT"] <- 1.5

cwd_wi_age_02[cwd_wi_age_02==""] <- 0.5

cwd_wi_age_02[cwd_wi_age_02=="F"] <- 0.5
table(cwd_wi_age_02)

cen_mat_wi_1 <- cbind(as.numeric(cwd_wi_age_1), as.numeric(cwd_test_event))
cen_mat_wi_2 <- cbind(as.numeric(cwd_wi_age_02), as.numeric(cwd_test_event02))
dim(cen_mat_wi_1)
dim(cen_mat_wi_2)

cen_mat_wi <- rbind(cen_mat_wi_1, cen_mat_wi_2)
dim(cen_mat_wi)
head(cen_mat_wi, 25)
tail(cen_mat_wi, 25)

#######################################################
#######################################################
#### Combine Survival Information for WI and Illinois
#######################################################
#######################################################

#### Create Variable for State, Management, year

dim(cen_mat)
dim(cen_mat_wi)

cen_mat_il_wi <- rbind(cen_mat, cen_mat_wi)
dim(cen_mat_il_wi)
head(cen_mat_il_wi)
tail(cen_mat_il_wi)
```

```
survdat_il_wi <- Surv(time = cen_mat_il_wi[,1],
                      event = cen_mat_il_wi[,2],
                      type='right',
                      origin=0)
table(survdat_il_wi)


# County ID at individual level for spatial analysis
counties <- c(as.character(cwd_il$HARVESTCO), as.character(cwd_wi$county),
              as.character(cwd_wi_add_on$County))

# Variables for formulat object in survival model

state_var <- c(rep("Illinois", times= dim(cen_mat)[1]),
               rep( "Wisconsin",times= dim(cen_mat_wi)[1]))

year_var <- c(cwd_il$FISCALYEAR, cwd_wi$year, cwd_wi_add_on$year)
# For illnois use GROUP variable
# For wisconsin use "pre-2007" vs "post_2007"
cwd_wi$management <- cwd_wi$year
cwd_wi$management[cwd_wi$year < 2008] <- "2001-2007 Management Period"
cwd_wi$management[cwd_wi$year > 2007 ] <- "2008-present Management Period"
table(cwd_wi$management)
table(cwd_il$GROUP)

cwd_wi_add_on$management <- cwd_wi_add_on$year
cwd_wi_add_on$management[cwd_wi_add_on$year < 2008] <- "2001-2007 Management
Period"
cwd_wi_add_on$management[cwd_wi_add_on$year > 2007 ] <- "2008-present Management
Period"
table(cwd_wi_add_on$management)
table(cwd_il$GROUP)

management_var <- c(as.character(cwd_il$GROUP), as.character(cwd_wi$management),
as.character(cwd_wi_add_on$management))

df_vars <- as.data.frame(cbind(state_var, year_var, management_var, counties))
names(df_vars)
head(df_vars)
tail(df_vars)

cen_mat_il_wi <- cen_mat_il_wi[-which(df_vars$counties == ""),]
survdat_il_wi <- survdat_il_wi[-which(df_vars$counties == ""),]
df_vars <- df_vars[-which(df_vars$counties == ""),]
df_vars$counties <- toupper(df_vars$counties)
df_vars$counties <- paste0(df_vars$counties, " ", "COUNTY")
# For now remove " ", "COUNTY"
df_vars$counties <- gsub(" COUNTY", "", df_vars$counties)

# Tiny bit of reformatting
# Put everything in one dataset before creating the survival object
df_vars$age <- cen_mat_il_wi[,1]
```

```
df_vars$status <- cen_mat_il_wi[,2]


head(df_vars)



########################################################
####  Total Harvest Data ################################
# Code to replicate Figures S1 and S2
library(ggplot2)
library(gridExtra)
setwd("/Users/andrewwhetten/Desktop/CWD_Case_Data/")
total_harvest_il <- read.csv("total_harvest_il_by_county_long.csv")
total_harvest_wi <- read.csv("wi_harvest.csv")

str(total_harvest_il)
str(total_harvest_wi)

wi_county_sub_list <- c("Kenosha", "Racine", "Waukesha","Jefferson",
                        "Walworth", "Rock", "Green","Lafayette",
                        "Grant", "Iowa", "Dane")

total_harvest_wi <- total_harvest_wi[total_harvest_wi$County %in% wi_county_sub_list,]
total_harvest_wi$Harvest <- total_harvest_wi$Total_Antlered +
total_harvest_wi$Total_Antlerless

df_il_total <- aggregate(Harvest~Year, total_harvest_il, sum)
df_wi_total_ant <- aggregate(Total_Antlered~Year, total_harvest_wi, sum)
df_wi_total_antless <- aggregate(Total_Antlerless~Year, total_harvest_wi, sum)

df_wi_total <- as.data.frame(cbind(df_wi_total_ant, df_wi_total_antless$Total_Antlerless))
df_wi_total$Harvest <- df_wi_total$Total_Antlered +
df_wi_total$`df_wi_total_antless$Total_Antlerless`

gg_il_county_harvest <- ggplot(data = total_harvest_il) + geom_line(aes(x=Year, y=Harvest,
color=County))
gg_il_total_harvest <- ggplot(data = df_il_total) + geom_line(aes(x=Year, y=Harvest))
gg_wi_county_harvest <- ggplot(data = total_harvest_wi) + geom_line(aes(x=Year, y=Harvest,
color=County))
gg_wi_total_harvest <- ggplot(data = df_wi_total) + geom_line(aes(x=Year, y=Harvest))

gg_wi_v_il_harvest <- ggplot() +
  geom_line(data = df_wi_total,aes(x=Year, y=Harvest,color="slateblue") ) +
  geom_line(data = df_il_total,aes(x=Year, y=Harvest,color= "maroon2")) +
  geom_segment(aes(x = 2007, y = 44243, xend = 2011, yend = 44243), color = "grey50",
linetype="dotted")+
  geom_segment(aes(x = 2011, y = 44243, xend = 2011, yend = 30919), color =
"grey50",linetype="dotted")+
  geom_segment(aes(x = 2007, y = 31644, xend = 2007, yend = 27325), color = "grey50",
linetype="dotted")+
  geom_segment(aes(x = 2007, y = 27325, xend = 2011, yend = 27325), color =
"grey50",linetype="dotted")+
  theme(text = element_text(family="Times",size=18),
```

```
        plot.title = element_text(size = 18),
        axis.text.x=element_text(size=18),
        panel.background = element_rect(fill = "white", colour = "grey50"),
        panel.grid.major = element_blank(),
        panel.grid.major.y=element_blank()) +
  scale_colour_manual(name = ' ',
                      values =c('slateblue'='slateblue','maroon2'='maroon2'), labels =
c('Illinois','Wisconsin'))
gg_wi_v_il_harvest

grid.arrange(gg_il_county_harvest, gg_wi_county_harvest, ncol=2)
grid.arrange(gg_il_total_harvest, gg_wi_total_harvest)


counties_select_wi <- c("Lafayette", "Walworth", "Rock", "Green")
counties_select_il <- c("McHenry", "Jodaviess", "Boone", "Stevenson",
                        "Winnebago")

# total_harvest_il

sub_il <-which(total_harvest_il$County %in% counties_select_il)
sub_wi <-which(total_harvest_wi$County %in% counties_select_wi)

border_harvest_il <- total_harvest_il[sub_il,]
border_harvest_wi <- total_harvest_wi[sub_wi,]


gg_wi_harvest <- ggplot() +
  geom_line(data = border_harvest_wi[border_harvest_wi$Year>2002,],aes(x=Year,
y=Harvest,color=County) ) +
  theme(text = element_text(family="Times",size=24),
        plot.title = element_text(size = 24),
        axis.text.x=element_text(size=24),
        panel.background = element_rect(fill = "white", colour = "grey50"),
        panel.grid.major = element_blank(),
        panel.grid.major.y=element_blank())+
  scale_color_brewer(palette="Dark2")

gg_il_harvest <- ggplot() +
  geom_line(data = border_harvest_il,aes(x=Year, y=Harvest,color=County) ) +
  theme(text = element_text(family="Times",size=24),
        plot.title = element_text(size = 24),
        axis.text.x=element_text(size=24),
        panel.background = element_rect(fill = "white", colour = "grey50"),
        panel.grid.major = element_blank(),
        panel.grid.major.y=element_blank())+ scale_color_brewer(palette="Dark2")


grid.arrange(gg_il_harvest, gg_wi_harvest, ncol=2)

  geom_line(data = df_il_total,aes(x=Year, y=Harvest,color= "maroon2")) +
  geom_segment(aes(x = 2007, y = 44243, xend = 2011, yend = 44243), color = "grey50",
linetype="dotted")+
```

```
  geom_segment(aes(x = 2011, y = 44243, xend = 2011, yend = 30919), color =
"grey50",linetype="dotted")+
  geom_segment(aes(x = 2007, y = 31644, xend = 2007, yend = 27325), color = "grey50",
linetype="dotted")+
  geom_segment(aes(x = 2007, y = 27325, xend = 2011, yend = 27325), color =
"grey50",linetype="dotted")+
  theme(text = element_text(family="Times",size=18),
        plot.title = element_text(size = 18),
        axis.text.x=element_text(size=18),
        panel.background = element_rect(fill = "white", colour = "grey50"),
        panel.grid.major = element_blank(),
        panel.grid.major.y=element_blank()) +
  scale_colour_manual(name = ' ',
                      values =c('slateblue'='slateblue','maroon2'='maroon2'), labels =
c('Illinois','Wisconsin'))
gg_wi_v_il_harvest




############################################################
############################################################
## Code to replicate component  of Figure 1 for Manuscript
############################################################
############################################################


library(usmap)
library(ggplot2)
library(RColorBrewer)
usmap::usmap_crs()
us_counties <- map_data("county")
class(us_counties)
head(us_counties)
wi_il_counties  <- us_counties[us_counties$region == "illinois" | us_counties$region ==
"wisconsin",]
head(wi_il_counties)

winnebago_wi_remove <- which(wi_il_counties$region == "wisconsin" &
                             wi_il_counties$subregion == "winnebago")
wi_il_counties <- wi_il_counties[-winnebago_wi_remove, ]
jefferson_il_remove <- which(wi_il_counties$region == "illinois" &
                             wi_il_counties$subregion == "jefferson")
wi_il_counties <- wi_il_counties[-jefferson_il_remove, ]



cases_03 <- as.numeric()
rel_freq_03 <- as.numeric()
for (i in 1:length(wi_il_counties$region)) {
  #i= 1957
  cnty_name <- wi_il_counties$subregion[i]
  cnty_name <- toupper(cnty_name)
  cnty_name <- gsub(" ","", cnty_name)
```

```
  case_tab <- table(df_vars$status[df_vars$counties == cnty_name &
as.numeric(df_vars$year_var) < 2008])

  cwd_test_03_pos <- case_tab[2]
  cwd_test_rel <- case_tab[2] / (case_tab[1]+case_tab[2])
  cases_03 <- c(cases_03, cwd_test_03_pos)
  rel_freq_03 <- c(rel_freq_03, cwd_test_rel)
}
wi_il_counties$cases03 <- cases_03
wi_il_counties$rel_freq03 <- rel_freq_03

counties_impt <- c("grant", "iowa", "lafayette", "green", "dane",
                   "rock", "walworth", "racine", "kenosha", "jefferson", "waukesha",
                   "jo daviess", "stephenson", "winnebago",
                   "boone", "mchenry", "lake", "carroll",
                   "ogle", "de kalb", "kane", "du page", "cook",
                   "whiteside", "lee", "la salle", "kendall",
                   "rock island", "henry", "bureau", "grundy", "will")
county_impt_id <- which(wi_il_counties$subregion %in% counties_impt)
wi_il_counties <- wi_il_counties[county_impt_id,]


wi_il_border_dat  <- us_counties[us_counties$region == "illinois" | us_counties$region ==
"wisconsin",]

us_states <- map_data("state")
us_states <- us_states[us_states$region == "wisconsin" | us_states$region == "illinois",]

gg_cases_02_08 <- ggplot() +
  geom_polygon(data = wi_il_counties,
             mapping = aes(x = long, y = lat,
                           group = group,  fill = cases03), size = 0.1) +
  coord_map(projection = "albers", lat0 = 39, lat1 = 45) +
  geom_polygon(data = us_states[us_states$region == "wisconsin",], mapping = aes(x = long, y
= lat,
                                                                   group = group), alpha = 0.1, color = "black")
+
  geom_polygon(data = us_states[us_states$region == "illinois",], mapping = aes(x = long, y =
lat,
                                                                  group = group), alpha = 0.1, color = "black")
+
  scale_fill_continuous(type = "viridis") +
  theme(axis.line=element_blank(),
        axis.text=element_blank(),
        axis.ticks=element_blank(),
        axis.title=element_blank(),
        panel.background=element_blank(),
        panel.border=element_blank(),
        panel.grid=element_blank())


#########################
# 2008-2022 visualization
```

```
us_counties <- map_data("county")
class(us_counties)
head(us_counties)
wi_il_counties2  <- us_counties[us_counties$region == "illinois" | us_counties$region ==
"wisconsin",]
head(wi_il_counties2)

winnebago_wi_remove <- which(wi_il_counties2$region == "wisconsin" &
                             wi_il_counties2$subregion == "winnebago")
wi_il_counties2 <- wi_il_counties2[-winnebago_wi_remove, ]
jefferson_il_remove <- which(wi_il_counties2$region == "illinois" &
                             wi_il_counties2$subregion == "jefferson")
wi_il_counties2 <- wi_il_counties2[-jefferson_il_remove, ]


cases_08 <- as.numeric()
rel_freq_08 <- as.numeric()
for (i in 1:length(wi_il_counties2$region)) {
  cnty_name <- wi_il_counties2$subregion[i]
  cnty_name <- toupper(cnty_name)
  cnty_name <- gsub(" ","", cnty_name)

  case_tab <- table(df_vars$status[df_vars$counties == cnty_name &
as.numeric(df_vars$year_var) >= 2008])

  cwd_test_08_pos <- case_tab[2]
  cwd_test_rel <- case_tab[2] / (case_tab[1]+case_tab[2])
  cases_08 <- c(cases_08, cwd_test_08_pos)
  rel_freq_08 <- c(rel_freq_08, cwd_test_rel)
}
wi_il_counties2$cases08 <- cases_08
wi_il_counties2$rel_freq08 <- rel_freq_08


counties_impt <- c("grant", "iowa", "lafayette", "green", "dane",
                   "rock", "walworth", "racine", "kenosha", "jefferson", "waukesha",
                   "jo daviess", "stephenson", "winnebago",
                   "boone", "mchenry", "lake", "carroll",
                   "ogle", "de kalb", "kane", "du page", "cook",
                   "whiteside", "lee", "la salle", "kendall",
                   "rock island", "henry", "bureau", "grundy", "will")
county_impt_id <- which(wi_il_counties2$subregion %in% counties_impt)
wi_il_counties2 <- wi_il_counties2[county_impt_id,]


wi_il_border_dat  <- us_counties[us_counties$region == "illinois" | us_counties$region ==
"wisconsin",]

us_states <- map_data("state")
us_states <- us_states[us_states$region == "wisconsin" | us_states$region == "illinois",]


gg_cases_08_22 <- ggplot() +
  geom_polygon(data = wi_il_counties2,
```

```
            mapping = aes(x = long, y = lat,
                        group = group,  fill = sqrt(cases08)), size = 0.1) +
  coord_map(projection = "albers", lat0 = 39, lat1 = 45) +
  geom_polygon(data = us_states[us_states$region == "wisconsin",], mapping = aes(x = long, y
= lat,
                                                   group = group), alpha = 0.1, color = "black")
+
  geom_polygon(data = us_states[us_states$region == "illinois",], mapping = aes(x = long, y =
lat,
                                                   group = group), alpha = 0.1, color = "black")
+
  scale_fill_continuous(type = "viridis") +
  theme(axis.line=element_blank(),
        axis.text=element_blank(),
        axis.ticks=element_blank(),
        axis.title=element_blank(),
        panel.background=element_blank(),
        panel.border=element_blank(),
        panel.grid=element_blank())



##########################################################
#### Case counts over time (by state and maybe by county)

table_cases_cty_mchenry <- table(as.numeric(df_vars$year_var), df_vars$status,
df_vars$counties=="MCHENRY")
table_cases_cty_mchenry <- table_cases_cty_mchenry[,,2]
table_cases_cty_wal <- table(as.numeric(df_vars$year_var), df_vars$status,
df_vars$counties=="WALWORTH")
table_cases_cty_wal <- table_cases_cty_wal[,,2]
rel_freq_mchenry <- table_cases_cty_mchenry[1:22,2]/(table_cases_cty_mchenry[1:22,1] +
table_cases_cty_mchenry[1:22,2])
rel_freq_wal <- table_cases_cty_wal[1:22,2]/(table_cases_cty_wal[1:22,1] +
table_cases_cty_wal[1:22,2])
rel_freq_mchenry <- as.data.frame(rel_freq_mchenry)
rel_freq_wal <- as.data.frame(rel_freq_wal)
rel_freq_wal[22,] <- NaN

cty_id_ex <- as.factor(rep(c("Walworth", "McHenry"), each = length(2001:2022)))

df_border_ex <-as.data.frame(cbind(c(2001:2022, 2001:2022),
                          c(rel_freq_wal$rel_freq_wal, rel_freq_mchenry$rel_freq_mchenry)))
df_border_ex$cty <- cty_id_ex

ggplot(data = df_border_ex,aes(x=V1, y=V2,color=df_border_ex$cty), ) +
  geom_point()

gg_relfreq_ex <- ggplot(data = df_border_ex) +
  geom_point(aes(x=V1, y=V2, color =cty)) +
  geom_line(aes(x=V1, y=V2, color=cty)) +
  scale_color_manual(values = c("aquamarine4","goldenrod3")) +
  theme(text = element_text(family="Times",size=18),
        plot.title = element_text(size = 18),
```

```
        axis.text.x=element_text(size=18),
        panel.background = element_rect(fill = "white", colour = "black"),
        panel.grid.major = element_blank(),
        panel.grid.major.y=element_blank(),
        legend.position =   "none") +
  geom_vline(xintercept = 2007, linetype= "dotted")+
  ylab("Relative Frequency (+) Tests") +
  xlab("Year")



table_cases <- table(as.numeric(df_vars$year_var), df_vars$status, df_vars$state_var)
il_cases_table <- table_cases[1:22, 1:2,1]
wi_cases_table <- table_cases[1:22, 1:2,2]


rel_freq_il <- il_cases_table[1:22,2]/(il_cases_table[1:22,1] + il_cases_table[1:22,2])
rel_freq_wi <- wi_cases_table[1:22,2]/(wi_cases_table[1:22,1] + wi_cases_table[1:22,2])

rel_freq_il <- as.data.frame(rel_freq_il)
rel_freq_wi <- as.data.frame(rel_freq_wi)

state_id_ex <- as.factor(rep(c("Illinois","Wisconsin"), each = length(2001:2022)))
df_rel_state <- as.data.frame(cbind(c(2001:2022, 2001:2022),c(rel_freq_il$rel_freq_il,
rel_freq_wi$rel_freq_wi)))
df_rel_state$state <- state_id_ex

gg_relfreq_cases <- ggplot(data = df_rel_state) +
  geom_point(aes(x=V1, y=V2, color =state)) +
  geom_line(aes(x=V1, y=V2, color=state)) +
  scale_color_manual(values = c("aquamarine4","goldenrod3")) +
  theme(text = element_text(family="Times",size=18),
        plot.title = element_text(size = 18),
        axis.text.x=element_text(size=18),
        panel.background = element_rect(fill = "white", colour = "black"),
        panel.grid.major = element_blank(),
        panel.grid.major.y=element_blank(),
        legend.position = "none") +
  geom_vline(xintercept = 2007, linetype= "dotted")+
  ylab("Relative Frequency (+) Tests") +
  xlab("Year")


# sharpshooting visual
head(df_vars)
table(df_vars$management_var)
df_vars_sharp_il <- df_vars[df_vars$management_var == "SHARPSHOOTING",]
head(df_vars_sharp_il)

table_cull_cty <- table(as.numeric(df_vars_sharp_il$year_var),
                        df_vars_sharp_il$counties == "MCHENRY")
mchenry_cull <- as.data.frame(table_cull_cty[,2])
names(mchenry_cull) <- "cull"
```

```
cull_subplot <- ggplot() +
  geom_point(data = mchenry_cull, aes(x=2003:2022, y=cull), color = "aquamarine4", size =2.5)
+
  geom_line(data = mchenry_cull, aes(x=2003:2022, y=cull),color = "aquamarine4", size =1.5) +
  theme(text = element_text(family="Times",size=48),
        plot.title = element_text(size = 48),
        axis.text.x=element_text(size=48),
        axis.text.y=element_text(size=48),
        panel.background = element_rect(fill = "white", colour = "black"),
        panel.grid.major = element_blank(),
        panel.grid.major.y=element_blank(),
        legend.position = "none") +
  geom_vline(xintercept = 2007, linetype= "dotted")+
  ylab("# of Deer Culled (McHenry)") +
  xlab(" ")



table_cases <- table(as.numeric(df_vars_sharp_il$year_var), df_vars_sharp_il$status)

rel_freq_sharp <- table_cases[1:20,2] / (table_cases[1:20,1] + table_cases[1:20,2])
sharp_total <- table_cases[1:20,1] + table_cases[1:20,2]

il_sharp_df <- as.data.frame(cbind(rel_freq_sharp, sharp_total))

gg_cull_yr_il <- ggplot() +
  geom_line( aes(x=2003:2022, y=sharp_total),color="aquamarine4", size = 1.5)+
  geom_point( aes(x=2003:2022, y=sharp_total),color="aquamarine4", size = 2.5)+
  geom_vline(xintercept = 2007, linetype= "dotted")+
  #geom_bar( aes(x=2003:2022,y=sharp_total), stat="identity", size=.1, fill="blue",
color="black", alpha=.4) +
  theme(text = element_text(family="Times",size=48),
        plot.title = element_text(size = 48),
        axis.text.x=element_text(size=48),
        axis.text.y=element_text(size=48),
        panel.background = element_rect(fill = "white", colour = "black"),
        panel.grid.major = element_blank(),
        panel.grid.major.y=element_blank(),
        legend.position = "none") + ylab("# of Deer Culled (IL)") + xlab("Year")
#
gg_blank <- ggplot() + theme(text = element_text(family="Times",size=48),
                             plot.title = element_text(size = 48),
                             axis.text.x=element_text(size=30),
                             panel.background = element_rect(fill = "white", colour = "white"),
                             panel.grid.major = element_blank(),
                             panel.grid.major.y=element_blank(),
                             legend.title = element_blank())

# A few different layouts and components of Figure 1
lay <- rbind(c(1,1,3,3,3,3),
             c(1,1,3,3,3,3),
             c(1,1,3,3,3,3))
grid.arrange(gg_cases_02_08, gg_cases_08_22, gg_relfreq_cases, layout_matrix = lay)
```

```
maps_grob <- grid.arrange(gg_cases_02_08, gg_cases_08_22)
line_plots_grob <- grid.arrange(gg_relfreq_cases, gg_relfreq_ex)
grid.arrange(maps_grob, line_plots_grob, ncol = 2)

grid.arrange(maps_grob, line_plots_grob, ncol = 2)
grid.arrange(maps_grob, line_plots_grob, layout_matrix = lay)
cull_subplot
gg_cull_yr_il



######################################################################
##### Add other Covariates to df_Vars dataframe ######################

# Var: Number of Deer Culled in previous year
head(df_vars)
num_culled_lag1 <- as.numeric()
for (i in 1:dim(df_vars)[1]) {
  temp_county <- df_vars$counties[i]
  temp_year <- as.numeric(df_vars$year_var[i])
  temp_find <- which(df_vars$counties %in% temp_county)
  df_vars_temp <- df_vars[temp_find,]
  culled_temp <- sum(df_vars_temp$management_var[df_vars_temp$year_var ==
                                           temp_year - 1 ] == "SHARPSHOOTING")
  num_culled_lag1 <- c(num_culled_lag1, culled_temp)
}

df_vars$number_culled_lag1 <- num_culled_lag1
tail(df_vars, 20)


# Var: All deer culled from 1-3 years ago in a county
num_culled_lag1_3 <- as.numeric()
for (i in 1:dim(df_vars)[1]) {
  #i=1
  temp_county <- df_vars$counties[i]
  temp_year <- as.numeric(df_vars$year_var[i])
  temp_find <- which(df_vars$counties %in% temp_county)
  df_vars_temp <- df_vars[temp_find,]
  # culled_temp <- sum(df_vars_temp$management_var[df_vars_temp$year_var ==
  #                                          temp_year - 3 ] == "SHARPSHOOTING")
  culled_temp <- sum(df_vars_temp$management_var[df_vars_temp$year_var == temp_year -
1 |
                                           df_vars_temp$year_var == temp_year - 2 |
                                           df_vars_temp$year_var == temp_year - 3] ==
"SHARPSHOOTING")
  num_culled_lag1_3 <- c(num_culled_lag1_3, culled_temp)
}

df_vars$number_culled_lag1_3 <- num_culled_lag1_3
tail(df_vars, 20)


# Var: All deer culled from 3-5 years ago in a county
```

```
num_culled_lag3_5 <- as.numeric()
for (i in 1:dim(df_vars)[1]) {
  #i=1
  temp_county <- df_vars$counties[i]
  temp_year <- as.numeric(df_vars$year_var[i])
  temp_find <- which(df_vars$counties %in% temp_county)
  df_vars_temp <- df_vars[temp_find,]
  # culled_temp <- sum(df_vars_temp$management_var[df_vars_temp$year_var ==
  #                                               temp_year - 3 ] == "SHARPSHOOTING")
  culled_temp <- sum(df_vars_temp$management_var[df_vars_temp$year_var == temp_year - 
3 |
                                              df_vars_temp$year_var == temp_year - 4 |
                                              df_vars_temp$year_var == temp_year - 5] == 
"SHARPSHOOTING")
  num_culled_lag3_5 <- c(num_culled_lag3_5, culled_temp)
}

df_vars$number_culled_lag3_5 <- num_culled_lag3_5
tail(df_vars, 20)

########################################################################
# Total Harvest Variables
names(total_harvest_il)
names(total_harvest_wi)
names(total_harvest_wi[,c(3,2,12)])
df_harvest <- as.data.frame(rbind(total_harvest_il, total_harvest_wi[,c(3,2,12)]))
head(df_harvest)
tail(df_harvest)
df_harvest$County <- toupper(df_harvest$County)
# Var 1: Number of Deer Culled in previous year
head(df_vars)
# df_vars[147693:147699,]

num_harvest_lag1 <- as.numeric()
for (i in 1:dim(df_vars)[1]) {
  #i=1
  temp_county <- df_vars$counties[i]
  temp_year <- as.numeric(df_vars$year_var[i])
  if(temp_county %in% df_harvest$County & temp_year %in% df_harvest$Year){
    temp_harvest <- df_harvest$Harvest[df_harvest$County == temp_county & df_harvest$Year 
== temp_year -1]
    if(length(temp_harvest) <1){temp_harvest = NA}
  }
  else{temp_harvest = NA}

  num_harvest_lag1 <- c(num_harvest_lag1, temp_harvest)
}

df_vars$number_harvest_lag1 <- num_harvest_lag1
tail(df_vars, 20)
head(df_vars, 20)
```

```
# Var: Harvest from 3 years ago in a county
num_harvest_lag3 <- as.numeric()
for (i in 1:dim(df_vars)[1]) {
  #i=1
  temp_county <- df_vars$counties[i]
  temp_year <- as.numeric(df_vars$year_var[i])
  if(temp_county %in% df_harvest$County & temp_year %in% df_harvest$Year){
    temp_harvest <- df_harvest$Harvest[df_harvest$County == temp_county & df_harvest$Year
== temp_year -3]
    if(length(temp_harvest) <1){temp_harvest = NA}
  }
  else{temp_harvest = NA}

  num_harvest_lag3 <- c(num_harvest_lag3, temp_harvest)
}

df_vars$number_harvest_lag3 <- num_harvest_lag3
tail(df_vars, 20)




# Var: Relative Frequency of Deer Culled in County relative to total deer culled in state
# from 1-3 years ago
num_culled_state_prop_lag1_3 <- as.numeric()
for (i in 1:dim(df_vars)[1]) {
  #i=1
  temp_county <- df_vars$counties[i]
  temp_year <- as.numeric(df_vars$year_var[i])
  #temp_year <- 2019
  temp_find <- which(df_vars$counties %in% temp_county)
  df_vars_temp <- df_vars[temp_find,]
  # culled_temp <- sum(df_vars_temp$management_var[df_vars_temp$year_var ==
  #                                              temp_year - 3 ] == "SHARPSHOOTING")
  culled_temp <- sum(df_vars_temp$management_var[df_vars_temp$year_var == temp_year -
1 |
                                              df_vars_temp$year_var == temp_year - 2 |
                                              df_vars_temp$year_var == temp_year - 3] ==
"SHARPSHOOTING")
  culled_temp_ttl <- sum(df_vars$management_var[df_vars_temp$year_var == temp_year - 1 |
                                             df_vars_temp$year_var == temp_year - 2 |
                                             df_vars_temp$year_var == temp_year - 3] ==
"SHARPSHOOTING")
  culled_temp_prop <- culled_temp/culled_temp_ttl
  num_culled_state_prop_lag1_3 <- c(num_culled_state_prop_lag1_3, culled_temp_prop)
}

df_vars$num_culled_state_prop_lag1_3 <- num_culled_state_prop_lag1_3
tail(df_vars, 20)


# Var: Relative Frequency of Deer Culled in County relative to total deer culled in state
# from 3-5 years ago
num_culled_state_prop_lag3_5 <- as.numeric()
```

```
for (i in 1:dim(df_vars)[1]) {
  #i=1
  temp_county <- df_vars$counties[i]
  temp_year <- as.numeric(df_vars$year_var[i])
  #temp_year <- 2019
  temp_find <- which(df_vars$counties %in% temp_county)
  df_vars_temp <- df_vars[temp_find,]
  culled_temp <- sum(df_vars_temp$management_var[df_vars_temp$year_var == temp_year -
3 |
                                              df_vars_temp$year_var == temp_year - 4 |
                                              df_vars_temp$year_var == temp_year - 5] ==
"SHARPSHOOTING")
  culled_temp_ttl <- sum(df_vars$management_var[df_vars_temp$year_var == temp_year - 3 |
                                             df_vars_temp$year_var == temp_year - 4 |
                                             df_vars_temp$year_var == temp_year - 5] ==
"SHARPSHOOTING")
  culled_temp_prop <- culled_temp/culled_temp_ttl
  num_culled_state_prop_lag3_5 <- c(num_culled_state_prop_lag3_5, culled_temp_prop)
}

df_vars$num_culled_state_prop_lag3_5 <- num_culled_state_prop_lag3_5
tail(df_vars, 20)



######################################################################
# Var: Number of positive cases in adjacent counties in previous year
wi_il_counties  <- us_counties[us_counties$region == "illinois" | us_counties$region ==
"wisconsin",]
head(wi_il_counties)

winnebago_wi_remove <- which(wi_il_counties$region == "wisconsin" &
                          wi_il_counties$subregion == "winnebago")
wi_il_counties <- wi_il_counties[-winnebago_wi_remove, ]
jefferson_il_remove <- which(wi_il_counties$region == "illinois" &
                          wi_il_counties$subregion == "jefferson")
wi_il_counties <- wi_il_counties[-jefferson_il_remove, ]
counties_impt <- c("grant", "iowa", "lafayette", "green", "dane",
                "rock", "walworth", "racine", "kenosha", "jefferson", "waukesha",
                "jo daviess", "stephenson", "winnebago",
                "boone", "mchenry", "lake", "carroll",
                "ogle", "de kalb", "kane", "du page", "cook",
                "whiteside", "lee", "la salle", "kendall",
                "rock island", "henry", "bureau", "grundy", "will")
county_impt_id <- which(wi_il_counties$subregion %in% counties_impt)
wi_il_counties <- wi_il_counties[county_impt_id,]

county_names <- names(table(wi_il_counties$subregion))

# "boone": "de kalb", "mcHenry", " "rock", "walworth", "winnebago"
#"bureau": "henry",  "la salle", "lee", "whiteside"
#"carroll": "jo daviess", "ogle","stephenson", "whiteside"
#"cook":  "du page", "lake"
#"dane":  "green", "iowa", "jefferson", "rock"
```

```
#de kalb": "boone, "kane","kendall","lee", "mchenry", "ogle", "la salle"
#"du page": "cook", "kane","kendall", "will"
#"grant": "iowa", "jo daviess", "lafayette"
# "green": "dane", "iowa",  "lafayette" , "rock","stephenson","winnebago"
# "grundy":  "kendall", "la salle", "will"

# "henry": "bureau", "rock island", "whiteside"
# "iowa": "dane", "grant", "green", "lafayette"
# "jefferson": "dane", "rock", "walworth", "waukesha"
# "jo daviess": "carroll", "grant", "lafayette", "stephenson"
# "kane": "cook", "de kalb", "du page", "kendall", "mchenry"
# "kendall": "de kalb", "kane", "la salle", "grundy", "will"
# "kenosha":   "lake", "mchenry", "racine", "walworth"
# "la salle": "bureau", "de kalb", "grundy", "kendall, "lee"
# "lafayette": "grant", "green", "jo daviess", "iowa", "stephenson"
# "lake": "cook", "kenosha", "mchenry"

# "lee": "bureau", "de kalb", "la salle", "ogle", "whiteside"
# "mchenry":"boone" , "cook", "de kalb", "kane", "kenosha", "lake", "walworth"
# "ogle": "carroll", "de kalb", "lee", "stephenson", "whiteside", "winnebago"
# "racine": "kenosha", "walworth", "waukesha"
# "rock": "boone", "dane", "green", "jefferson", "walworth", "winnebago"
# "rock island": "henry","whiteside"
# "stephenson": "carroll", "green", "jo daviess", "lafayette", "ogle", "winnebago"
# "walworth": "boone", "jefferson", "kenosha","mchenry",  "racine", "rock", "waukesha"
# "waukesha": "jefferson", "racine", "walworth",
# "whiteside": "bureau",  "carroll", "henry",  "lee", "ogle"
# "will": "cook", "du page", "grundy", "kendall"
# "winnebago": "boone", "green", "rock", "stephenson", "ogle"

county_adj_list <- vector(mode = "list", length = 32)
county_adj_list[[1]] <- c("de kalb", "mcHenry", "rock", "walworth", "winnebago")
county_adj_list[[2]] <- c("henry",  "la salle", "lee", "whiteside")
county_adj_list[[3]] <- c("jo daviess", "ogle","stephenson", "whiteside")
county_adj_list[[4]] <- c("du page", "lake")
county_adj_list[[5]] <- c("green", "iowa", "jefferson", "rock")
county_adj_list[[6]] <- c("boone", "kane","kendall","lee", "mchenry", "ogle", "la salle")
county_adj_list[[7]] <- c("cook", "kane","kendall", "will" )
county_adj_list[[8]] <- c("iowa", "jo daviess", "lafayette")
county_adj_list[[9]] <- c("dane", "iowa",  "lafayette" , "rock","stephenson","winnebago")
county_adj_list[[10]] <- c("kendall", "la salle", "will")

county_adj_list[[11]] <- c("bureau", "rock island", "whiteside")
county_adj_list[[12]] <- c("dane", "grant", "green", "lafayette")
county_adj_list[[13]] <- c("dane", "rock", "walworth", "waukesha")
county_adj_list[[14]] <- c("carroll", "grant", "lafayette", "stephenson" )
county_adj_list[[15]] <- c("cook", "de kalb", "du page", "kendall", "mchenry" )
county_adj_list[[16]] <- c("de kalb", "kane", "la salle", "grundy", "will")
county_adj_list[[17]] <- c("lake", "mchenry", "racine", "walworth")
county_adj_list[[18]] <- c("bureau", "de kalb", "grundy", "kendall", "lee")
county_adj_list[[19]] <- c("grant", "green", "jo daviess", "iowa", "stephenson")
county_adj_list[[20]] <- c("cook", "kenosha", "mchenry")

county_adj_list[[21]] <- c("bureau", "de kalb", "la salle", "ogle", "whiteside" )
```

```
county_adj_list[[22]] <- c("boone" , "cook", "de kalb", "kane", "kenosha", "lake", "walworth")
county_adj_list[[23]] <- c("carroll", "de kalb", "lee", "stephenson", "whiteside", "winnebago")
county_adj_list[[24]] <- c("kenosha", "walworth", "waukesha")
county_adj_list[[25]] <- c("boone", "dane", "green", "jefferson", "walworth", "winnebago" )
county_adj_list[[26]] <- c("henry","whiteside")
county_adj_list[[27]] <- c("carroll", "green", "jo daviess", "lafayette", "ogle", "winnebago")
county_adj_list[[28]] <- c("boone", "jefferson", "kenosha","mchenry",  "racine", "rock",
"waukesha")
county_adj_list[[29]] <- c("jefferson", "racine", "walworth")
county_adj_list[[30]] <- c("bureau",  "carroll", "henry",  "lee", "ogle" )
county_adj_list[[31]] <- c("cook", "du page", "grundy", "kendall")
county_adj_list[[32]] <- c("boone", "green", "rock", "stephenson", "ogle")

adj_mat <- matrix(data = 0, nrow = 32, ncol = 32)

diag(adj_mat) <- 1

for (i in 1:32) {
  print(county_adj_list[[i]])
  temp <- which(county_names %in% county_adj_list[[i]])
  print(temp)
  adj_mat[i, temp] <- 1
}

county_names <- gsub(" ", "", county_names)
county_names <- toupper(county_names)

rownames(adj_mat) <- county_names
colnames(adj_mat) <- county_names


dim(adj_mat)
diag(adj_mat) <- rep(0, times=32)

# Write for loop to count number of positive cases in all
#adjacent counties for a given year
head(df_vars)
all_pos_adj_counties <- as.numeric()
for (i in 1:dim(df_vars)[1]) {
  temp_county <- df_vars$counties[i]
  temp_year <- as.numeric(df_vars$year_var[i])
  county_loc <-which(rownames(adj_mat) == temp_county)
  county_temp <- which(adj_mat[,county_loc]==1)
  adj_county_names_temp <- colnames(adj_mat[,county_temp])
  temp_find_adj <- which(df_vars$counties %in% adj_county_names_temp)
  df_vars_temp <- df_vars[temp_find_adj,]
  pos_cases_temp <- sum(df_vars_temp$status[df_vars_temp$year_var == temp_year - 1 ])
  all_pos_adj_counties <- c(all_pos_adj_counties, pos_cases_temp)
}

df_vars$number_cases_adj <- all_pos_adj_counties
tail(df_vars, 20)

# Var: Number of positive cases in adjacent counties in previous 3 years
```

```
# Write for loop to count number of positive cases in all
#adjacent counties for past 3 years
head(df_vars)
all_pos_adj_counties_3yrs <- as.numeric()
for (i in 1:dim(df_vars)[1]) {
  temp_county <- df_vars$counties[i]
  temp_year <- as.numeric(df_vars$year_var[i])
  county_loc <-which(rownames(adj_mat) == temp_county)
  county_temp <- which(adj_mat[,county_loc]==1)
  adj_county_names_temp <- colnames(adj_mat[,county_temp])
  temp_find_adj <- which(df_vars$counties %in% adj_county_names_temp)
  df_vars_temp <- df_vars[temp_find_adj,]
  pos_cases_temp <- sum(df_vars_temp$status[df_vars_temp$year_var == temp_year - 1 |
                                            df_vars_temp$year_var == temp_year - 2 |
                                            df_vars_temp$year_var == temp_year - 3])
  all_pos_adj_counties_3yrs <- c(all_pos_adj_counties_3yrs, pos_cases_temp)
}

df_vars$number_cases_adj_wthin_3 <- all_pos_adj_counties_3yrs
tail(df_vars, 20)

# Var: Number of positive cases in adjacent counties in previous 3 years
# Write for loop to count number of positive cases in all
#adjacent counties for past 3-5 years
head(df_vars)
all_pos_adj_counties_5yrs <- as.numeric()
for (i in 1:dim(df_vars)[1]) {
  #i=1
  temp_county <- df_vars$counties[i]
  temp_year <- as.numeric(df_vars$year_var[i])
  #temp_year <- 2021
  county_loc <-which(rownames(adj_mat) == temp_county)
  county_temp <- which(adj_mat[,county_loc]==1)
  adj_county_names_temp <- colnames(adj_mat[,county_temp])
  temp_find_adj <- which(df_vars$counties %in% adj_county_names_temp)
  df_vars_temp <- df_vars[temp_find_adj,]
  pos_cases_temp <- sum(df_vars_temp$status[df_vars_temp$year_var == temp_year - 3 |
                                            df_vars_temp$year_var == temp_year - 4 |
                                            df_vars_temp$year_var == temp_year - 5])
  all_pos_adj_counties_5yrs <- c(all_pos_adj_counties_5yrs, pos_cases_temp)
}

df_vars$number_cases_adj_wthin_3_5 <- all_pos_adj_counties_5yrs
tail(df_vars, 20)


# Var: Number of deer culled in all adjacent counties in previous year.

head(df_vars)
all_culled_adj_counties <- as.numeric()
for (i in 1:dim(df_vars)[1]) {
  #i=1
  temp_county <- df_vars$counties[i]
  temp_year <- as.numeric(df_vars$year_var[i])
```

```
  #temp_year <- 2019
  county_loc <-which(rownames(adj_mat) == temp_county)
  county_temp <- which(adj_mat[,county_loc]==1)
  adj_county_names_temp <- colnames(adj_mat[,county_temp])
  temp_find_adj <- which(df_vars$counties %in% adj_county_names_temp)
  df_vars_temp <- df_vars[temp_find_adj,]
  culled_temp <- sum(df_vars_temp$management_var[df_vars_temp$year_var ==
                                        temp_year - 1 ] == "SHARPSHOOTING")
  all_culled_adj_counties <- c(all_culled_adj_counties, culled_temp)
}

df_vars$number_culled_adj <- all_culled_adj_counties
tail(df_vars, 20)

# Var: Number of deer culled in all adjacent counties in previous 3 years.
head(df_vars)
all_culled_adj_counties_wthn_3 <- as.numeric()
for (i in 1:dim(df_vars)[1]) {
  temp_county <- df_vars$counties[i]
  temp_year <- as.numeric(df_vars$year_var[i])
  county_loc <-which(rownames(adj_mat) == temp_county)
  county_temp <- which(adj_mat[,county_loc]==1)
  adj_county_names_temp <- colnames(adj_mat[,county_temp])
  temp_find_adj <- which(df_vars$counties %in% adj_county_names_temp)
  df_vars_temp <- df_vars[temp_find_adj,]
  culled_temp <- sum(df_vars_temp$management_var[df_vars_temp$year_var == temp_year -
1 |
                                        df_vars_temp$year_var == temp_year - 2 |
                                        df_vars_temp$year_var == temp_year - 3
  ] == "SHARPSHOOTING")
  all_culled_adj_counties_wthn_3 <- c(all_culled_adj_counties_wthn_3, culled_temp)
}

df_vars$number_culled_adj_wthn_3 <- all_culled_adj_counties_wthn_3
tail(df_vars, 20)



# Var: Number of deer culled in all adjacent counties 3-5 yrs ago.
head(df_vars)
all_culled_adj_counties_lag3_5 <- as.numeric()
for (i in 1:dim(df_vars)[1]) {
  temp_county <- df_vars$counties[i]
  temp_year <- as.numeric(df_vars$year_var[i])
  county_loc <-which(rownames(adj_mat) == temp_county)
  county_temp <- which(adj_mat[,county_loc]==1)
  adj_county_names_temp <- colnames(adj_mat[,county_temp])
  temp_find_adj <- which(df_vars$counties %in% adj_county_names_temp)
  df_vars_temp <- df_vars[temp_find_adj,]
  culled_temp <- sum(df_vars_temp$management_var[df_vars_temp$year_var == temp_year -
3 |
                                        df_vars_temp$year_var == temp_year - 4 |
                                        df_vars_temp$year_var == temp_year - 5
  ] == "SHARPSHOOTING")
```

```
  all_culled_adj_counties_lag3_5 <- c(all_culled_adj_counties_lag3_5, culled_temp)
}

df_vars$all_culled_adj_counties_lag3_5 <- all_culled_adj_counties_lag3_5
tail(df_vars, 20)


#### Var: Harvest in all adjacent counties in previous year.

head(df_vars)
harvest_adj_counties_lag1 <- as.numeric()
for (i in 1:dim(df_vars)[1]) {
  temp_county <- df_vars$counties[i]
  temp_year <- as.numeric(df_vars$year_var[i])
  county_loc <-which(rownames(adj_mat) == temp_county)
  county_temp <- which(adj_mat[,county_loc]==1)
  adj_county_names_temp <- colnames(adj_mat[,county_temp])
  adj_harv_temp <- sum(df_harvest$Harvest[df_harvest$County %in%
                                           adj_county_names_temp &
                                           df_harvest$Year == temp_year -1])

  if(adj_harv_temp ==0){adj_harv_temp = NA}

  harvest_adj_counties_lag1 <- c(harvest_adj_counties_lag1, adj_harv_temp)
}

df_vars$harvest_adj_counties_lag1 <- harvest_adj_counties_lag1
tail(df_vars, 20)
# hist(df_vars$harvest_adj_counties_lag1)
table(df_vars$harvest_adj_counties_lag1 <10)

#Var: Harvest in all adjacent counties 3 years ago

head(df_vars)
harvest_adj_counties_lag3 <- as.numeric()
for (i in 1:dim(df_vars)[1]) {
  temp_county <- df_vars$counties[i]
  temp_year <- as.numeric(df_vars$year_var[i])
  county_loc <-which(rownames(adj_mat) == temp_county)
  county_temp <- which(adj_mat[,county_loc]==1)
  adj_county_names_temp <- colnames(adj_mat[,county_temp])
  adj_harv_temp <- sum(df_harvest$Harvest[df_harvest$County %in%
adj_county_names_temp &
                                           df_harvest$Year == temp_year -3])
  if(adj_harv_temp ==0){adj_harv_temp = NA}
  harvest_adj_counties_lag3 <- c(harvest_adj_counties_lag3, adj_harv_temp)
}

df_vars$harvest_adj_counties_lag3 <- harvest_adj_counties_lag3
tail(df_vars, 20)
```

```
# Var: Number of Kilometers of border along the WI-IL stateline

# Jo Davies: 58.57km
# Stephenson: 43.01km
# Winnebago: 37.79km
# Boone: 10.84 + 3.73 + 4.53 = 19.10km
# McHenry: 3.88 + 17.89 + 14.02 +1.73 + 3.36 + 0.78 = 41.66km
# Lake: 8.02 + 14.93 + 9.72 = 32.67km

# Kenosha: 9.72 + 22.88 + 8.84 = 41.44
# Walworth: 9.92 + 28.71 = 38.63
# Rock: 28.09 + 20.19 = 48.28
# Green: 14.94 + 14.69 = 29.63
#Lafayette: 9.44 + 11.95 + 26.90 = 48.29
# Grant: 17.52

border_county_name <- c("BOONE", "GRANT", "GREEN", "JODAVIESS", "KENOSHA",
                        "LAFAYETTE", "LAKE", "MCHENRY", "ROCK", "STEPHENSON",
                        "WALWORTH", "WINNEBAGO")
border_county_distance <- c(19.10, 17.52, 29.63, 58.57, 41.44,
                            48.29, 32.67, 41.66, 48.28, 43.01,
                            38.63, 37.79)
# ttl border distance ~233km
df_border_dist <-  as.data.frame(cbind(border_county_name, border_county_distance))
df_border_dist$border_county_distance <-
as.numeric(df_border_dist$border_county_distance)
head(df_vars)
border_length <- as.numeric()
for (i in 1:dim(df_vars)[1]) {
  #i=1
  temp_county <- df_vars$counties[i]
  #temp_county <- "WINNEBAGO"
  county_loc <-which(df_border_dist$border_county_name == temp_county)
  if(length(county_loc) < 1) { border_length <- c(border_length, 0)}
  else{border_length <- c(border_length, df_border_dist$border_county_distance[county_loc])}
}

df_vars$border_length <- border_length
tail(df_vars, 20)




##########################################################
##########################################################
table(as.numeric(df_vars$year_var), df_vars$num_culled_state_prop_lag1_3 > 0,
df_vars$counties)
table(as.numeric(df_vars$year_var), df_vars$num_culled_state_prop_lag1_3 > 0,
df_vars$counties == "BOONE")

table(df_vars$year_var[df_vars$counties =="BOONE"],
df_vars$num_culled_state_prop_lag1_3[df_vars$counties =="BOONE"])
plot(df_vars$year_var, )
```

```
########################################################
########################################################

# Final spatial variable... county cetroids
# Read in County Shapefiles for IL and WI
# as SpatialPolygonDataframes
library(maptools)

setwd("/Users/andrewwhetten/Desktop/KState Research/CWD_Case_Data/counties_US")
P4S.latlon <- CRS("+proj=longlat +datum=WGS84")
shape_wi_sect <- readShapePoly("County.shp", proj4string=P4S.latlon)
class(shape_wi_sect)
#str(shape_wi_sect[1])
plot(shape_wi_sect[shape_wi_sect$State == "Wisconsin" | shape_wi_sect$State == "Illinois",])
wi_il_shape <- shape_wi_sect[shape_wi_sect$State == "Wisconsin" | shape_wi_sect$State ==
"Illinois",]
# We could subset this shapefile further if needed
str(wi_il_shape$NAME)
wi_il_shape$NAME <- toupper(wi_il_shape$NAME)


library(rgeos)
centroids_wi_il_counties <- gCentroid(wi_il_shape, byid=TRUE)
plot(centroids_wi_il_counties)
class(centroids_wi_il_counties)
centroids_wi_il_counties
centroids_df <- as.data.frame(centroids_wi_il_counties)
centroids_df$counties <- wi_il_shape$NAME
head(centroids_df)
test_temp <- centroids_df[centroids_df$counties == "KENOSHA COUNTY" |
                    centroids_df$counties == "RACINE COUNTY" |
                    centroids_df$counties == "ROCK COUNTY" |
                    centroids_df$counties == "WALWORTH COUNTY" |
                    centroids_df$counties == "BOONE COUNTY" ,]
plot(test_temp$x, test_temp$y)


head(df_vars)


# remove WI's winnebago county
centroids_df[101,]
centroids_df[173,]
centroids_df <- centroids_df[-173,]

centroids_df$counties <- gsub(" ", "", centroids_df$counties, fixed = TRUE)
centroids_df$counties <- gsub("COUNTY", "", centroids_df$counties, fixed = TRUE)
centroids_df$counties <- gsub(".", "", centroids_df$counties, fixed = TRUE)
df_vars$counties <- gsub(" ", "", df_vars$counties, fixed = TRUE)


cty_coord_lon <- as.numeric()
cty_coord_lat <- as.numeric()
# it was 150161 before
```

```
for (i in 1:150169) {
  #i=352
  #i=1
  cty_id_temp <- df_vars$counties[i]
  loc_cty_temp <- which(centroids_df$counties == cty_id_temp)
  #if(length(loc_cty_temp) > 1){print(i)}
  cty_coord_lon <- c(cty_coord_lon,centroids_df$x[loc_cty_temp[1]])
  cty_coord_lat <- c(cty_coord_lat, centroids_df$y[loc_cty_temp[1]])
}
for (i in 150170:length(df_vars$counties)) {
  #i=93
  cty_id_temp <- df_vars$counties[i]
  loc_cty_temp <- which(centroids_df$counties == cty_id_temp)
  if(length(loc_cty_temp) > 1){
    cty_coord_lon <- c(cty_coord_lon,centroids_df$x[loc_cty_temp[2]])
    cty_coord_lat <- c(cty_coord_lat, centroids_df$y[loc_cty_temp[2]])
  }
  else{
    cty_coord_lon <- c(cty_coord_lon,centroids_df$x[loc_cty_temp[1]])
    cty_coord_lat <- c(cty_coord_lat, centroids_df$y[loc_cty_temp[1]])
  }

}
df_vars$county_centroid_lon <- cty_coord_lon
df_vars$county_centroid_lat <- cty_coord_lat

head(df_vars)
```

```
library(geosphere)
library(leaflet)
library(lubridate)
library(spatsurv)
library(survival)
library(sf)
library(sp)
library(gridExtra)
library(ggplot2)
library(gridExtra)

setwd("/Users/andrewwhetten/Desktop/CWD_Case_Data/")

df_vars <- read.csv("cwd_surv_data.csv")
head(df_vars)
str(df_vars)
############################################################
############ Fit Survival Random Forests Model ##############
############################################################
library(randomForest)
library(randomForestSRC)
library(pdp)
library(ggRandomForests)


# convert character vars to factor
df_vars$state_var <- as.factor(df_vars$state_var)
df_vars$year_var <- as.numeric(df_vars$year_var)
df_vars$management_var <- as.factor(df_vars$management_var)
df_vars$counties <- as.factor(df_vars$counties)

df_vars$num_culled_state_prop_lag1_3[is.nan(df_vars$num_culled_state_prop_lag1_3)] <- 0
#df_vars$num_culled_state_prop_lag2_4[is.nan(df_vars$num_culled_state_prop_lag2_4)] <- 0
df_vars$num_culled_state_prop_lag3_5[is.nan(df_vars$num_culled_state_prop_lag3_5)] <- 0
str(df_vars)
plot(df_vars$year_var, df_vars$num_culled_state_prop_lag1_3)
# df_vars_temp <- df_vars
#
df_vars_temp$num_culled_state_prop_lag1_3[is.nan(df_vars_temp$num_culled_state_prop_lag
1_3)] <- 0
# str(df_vars_temp)


# trial run... trying multiple adjustments to reduce computation time
# change columns again below
names(df_vars)
head(df_vars)
cor(df_vars[,c(7:16,18:21)])
names(df_vars[,c(1:11,13:14,16,18:21)])
###
# NOTE!!! NEXT TIME THIS IS RUN the removed columns need to be changed!
startTime <- Sys.time()
set.seed(2649)
# srf <- rfsrc(Surv(age, status)~., data = df_vars[,-c(7,8,14,17,19)], ntree = 50,
```

```
#             importance = TRUE, save.memory = TRUE, nodesize = 80)
srf <- rfsrc(Surv(age, status)~., data = df_vars[,c(1:11,13:14,16,18:21)], ntree = 50,
           importance = TRUE, save.memory = TRUE, nodesize = 80)
endTime <- Sys.time()
endTime - startTime

startTime <- Sys.time()
set.seed(2649)
# srf <- rfsrc(Surv(age, status)~., data = df_vars[,-c(7,8,14,17,19)], ntree = 50,
#             importance = TRUE, save.memory = TRUE, nodesize = 80)
srf <- rfsrc(Surv(age, status)~., data = df_vars[,2:25], ntree = 50,
           importance = TRUE, save.memory = TRUE, nodesize = 80)
endTime <- Sys.time()
endTime - startTime

cor(df_vars[,c(2,7:11,13:14,16,18:21)])

# RF using all variables (including those inducing high collinearity)
# startTime <- Sys.time()
# set.seed(2649)
# srf <- rfsrc(Surv(age, status)~., data = df_vars, ntree = 50,
#             importance = TRUE, save.memory = TRUE, nodesize = 80)
# endTime <- Sys.time()
# endTime - startTime


# Run time with 10 trees: 20.3 minutes
# Run time with 100 trees: ~3.5 hours

plot(gg_vimp(srf))


str(srf)
surv.oob <- srf$survival.oob
surv.oob <- srf$survival
timesrf <- srf$time.interest

df_vars_surv <- as.data.frame(cbind(df_vars,surv.oob))

df_vars_surv_wi_01_07 <- df_vars_surv[df_vars_surv$state == "Wisconsin" &
df_vars_surv$year_var < 2008,]
df_vars_surv_wi_08_22 <- df_vars_surv[df_vars_surv$state == "Wisconsin" &
df_vars_surv$year_var >= 2008,]
df_vars_surv_il_03_16 <- df_vars_surv[df_vars_surv$state == "Illinois"& df_vars_surv$year_var
< 2017,]
df_vars_surv_il_17_22 <- df_vars_surv[df_vars_surv$state == "Illinois"& df_vars_surv$year_var
>= 2017,]

surv_mean_wi_01_07 <- colMeans(df_vars_surv_wi_01_07[,c(26:35)])
surv_mean_wi_08_22 <- colMeans(df_vars_surv_wi_08_22[,c(26:35)])
surv_mean_il_03_16 <- colMeans(df_vars_surv_il_03_16[,c(26:35)])
surv_mean_il_17_22 <- colMeans(df_vars_surv_il_17_22[,c(26:35)])
```

```
surv_se_wi_01_07 <- as.numeric()
surv_se_wi_08_22 <- as.numeric()
surv_se_il_03_16 <- as.numeric()
surv_se_il_17_22 <- as.numeric()
for (i in 26:35) {
  surv_se_wi_01_07 <- c(surv_se_wi_01_07, sd(df_vars_surv_wi_01_07[,i])/
sqrt(dim(df_vars_surv_wi_01_07)[1]))
  surv_se_wi_08_22 <- c(surv_se_wi_08_22, sd(df_vars_surv_wi_08_22[,i])/
sqrt(dim(df_vars_surv_wi_08_22)[1]))
  surv_se_il_03_16 <- c(surv_se_il_03_16, sd(df_vars_surv_il_03_16[,i])/
sqrt(dim(df_vars_surv_il_03_16)[1]))
  surv_se_il_17_22 <- c(surv_se_il_17_22,sd(df_vars_surv_il_17_22[,i])/
sqrt(dim(df_vars_surv_il_17_22)[1]))
}


surv_means_states <- c(as.numeric(surv_mean_il_03_16),
                       as.numeric(surv_mean_il_17_22),
                       as.numeric(surv_mean_wi_01_07),
                       as.numeric(surv_mean_wi_08_22)
)
surv_se_states <- c(as.numeric(surv_se_il_03_16),
                    as.numeric(surv_se_il_17_22),
                    as.numeric(surv_se_wi_01_07),
                    as.numeric(surv_se_wi_08_22)
)
state_labels <- c(rep('IL: 2002-2016',times = 10),
                  rep('IL: 2017-2022',times = 10),
                  rep('WI: 2001-2007',times = 10),
                  rep('WI: 2008-2021',times = 10)
)
df_surv_mean_states <- as.data.frame(cbind(surv_means_states,surv_se_states, state_labels,
                                           rep(timesrf, times =4)))
names(df_surv_mean_states)
gg_surv_by_states <- ggplot() +
  geom_line(data = df_surv_mean_states,
            aes(x=as.numeric(V4), y= 1- as.numeric(surv_means_states), color = state_labels),
linewidth = 1.2) +
  # geom_ribbon(data = df_surv_mean_states,
  #             aes(x=as.numeric(V4), fill = state_labels,
  #                 ymin = (1- as.numeric(surv_means_states)) - 3*as.numeric(surv_se_states),
  #                 ymax = (1- as.numeric(surv_means_states))+ 3*as.numeric(surv_se_states)), alpha
= 0.2)+
  scale_color_manual(values=c("maroon2", "maroon4","steelblue1","steelblue4")) +
  # scale_fill_manual(values=c("maroon2", "maroon4","steelblue1","steelblue4")) +
  theme(text = element_text(family="Times",size=36),
        plot.title = element_text(size = 36),
        axis.text.x=element_text(size=36),
        panel.background = element_rect(fill = "white", colour = "black"),
        panel.grid.major = element_blank(),
        panel.grid.major.y=element_blank(),
        legend.position  = "right",
        legend.title = element_blank()) +
```

```
  ylab("Probability CWD (+)") + xlab("Age")





##########################################################################
#
chf_cov_df<- as.data.frame(cbind(df_vars, rowMeans(srf$survival)))

names(chf_cov_df)[26] <- "Survival"
#plot(wi_il_shape)

head(chf_cov_df)

library(dplyr)

rock_cty_chf <- chf_cov_df[chf_cov_df$counties == "ROCK",]
green_cty_chf <- chf_cov_df[chf_cov_df$counties == "GREEN",]
walworth_cty_chf <- chf_cov_df[chf_cov_df$counties == "WALWORTH",]
lafay_cty_chf <- chf_cov_df[chf_cov_df$counties == "LAFAYETTE",]



winnebago_cty_chf <- chf_cov_df[chf_cov_df$counties == "WINNEBAGO",]
boone_cty_chf <- chf_cov_df[chf_cov_df$counties == "BOONE",]
mchenry_cty_chf <- chf_cov_df[chf_cov_df$counties == "MCHENRY",]
stephenson_cty_chf <- chf_cov_df[chf_cov_df$counties == "STEPHENSON",]
jodaviess_cty_chf <- chf_cov_df[chf_cov_df$counties == "JODAVIESS",]


rock_cty_chf_sum <- rock_cty_chf %>%
  group_by(year_var) %>%
  summarise(year_chf = mean(Survival), sharp = sum())
green_cty_chf_sum <- green_cty_chf %>%
  group_by(year_var) %>%
  summarise(year_chf = mean(Survival))
walworth_cty_chf_sum <- walworth_cty_chf %>%
  group_by(year_var) %>%
  summarise(year_chf = mean(Survival))
lafay_cty_chf_sum <- lafay_cty_chf %>%
  group_by(year_var) %>%
  summarise(year_chf = mean(Survival))


winnebago_cty_chf_sum <- winnebago_cty_chf %>%
  group_by(year_var) %>%
  summarise(year_chf = mean(Survival), sharp = sum(management_var ==
"SHARPSHOOTING"))
boone_cty_chf_sum <- boone_cty_chf %>%
  group_by(year_var) %>%
  summarise(year_chf = mean(Survival), sharp = sum(management_var ==
"SHARPSHOOTING"))
```

```
mchenry_cty_chf_sum <- mchenry_cty_chf %>%
  group_by(year_var) %>%
  summarise(year_chf = mean(Survival), sharp = sum(management_var ==
"SHARPSHOOTING"))

stephenson_cty_chf_sum <- stephenson_cty_chf %>%
  group_by(year_var) %>%
  summarise(year_chf = mean(Survival), sharp = sum(management_var ==
"SHARPSHOOTING"))

jodaviess_cty_chf_sum <- jodaviess_cty_chf %>%
  group_by(year_var) %>%
  summarise(year_chf = mean(Survival), sharp = sum(management_var ==
"SHARPSHOOTING"))


# plot(jodaviess_cty_chf_sum$year_var, jodaviess_cty_chf_sum$sharp)
# plot(winnebago_cty_chf_sum$year_var, winnebago_cty_chf_sum$year_chf, type = "b",
#      col = "blue", ylim = c(0.5,1))
# lines(boone_cty_chf_sum$year_var, boone_cty_chf_sum$year_chf, type = "b", col =
"lightblue")
# lines(mchenry_cty_chf_sum$year_var, mchenry_cty_chf_sum$year_chf, type = "b", col =
"purple")
# lines(jodaviess_cty_chf_sum$year_var, jodaviess_cty_chf_sum$year_chf, type = "b", col =
"darkblue")
# lines(stephenson_cty_chf_sum$year_var, stephenson_cty_chf_sum$year_chf, type = "b", col
= "darkred")
#
# plot(winnebago_cty_chf_sum$year_var, winnebago_cty_chf_sum$sharp, type = "b",
#      col = "blue", ylim = c(0,600))
# lines(boone_cty_chf_sum$year_var, boone_cty_chf_sum$sharp, type = "b", col = "lightblue")
# lines(mchenry_cty_chf_sum$year_var, mchenry_cty_chf_sum$sharp, type = "b", col =
"purple")
# lines(jodaviess_cty_chf_sum$year_var, jodaviess_cty_chf_sum$sharp, type = "b", col =
"darkblue")
# lines(stephenson_cty_chf_sum$year_var, stephenson_cty_chf_sum$sharp, type = "b", col =
"darkred")

df_il_county_surv_sharp <- as.data.frame(rbind(winnebago_cty_chf_sum, boone_cty_chf_sum,
mchenry_cty_chf_sum,
                                     jodaviess_cty_chf_sum, stephenson_cty_chf_sum))
df_wi_county_surv_sharp <- as.data.frame(rbind(rock_cty_chf_sum[,c(1,2)],
green_cty_chf_sum, walworth_cty_chf_sum,
                                     lafay_cty_chf_sum))

df_il_county_surv_sharp$County <- rep(c("Winnebago","Boone","McHenry","Jo
Daviess","Stephenson"), each =20)
df_wi_county_surv_sharp$County <- rep(c("Rock", "Green", "Walworth", "LaFayette"), times =
c(21,21,21,20))


gg_il_surv <- ggplot() +
  geom_smooth(data = df_il_county_surv_sharp, aes(x=year_var, y=1-year_chf, color=County),
alpha = 0.85, se = FALSE) +
```

```
  #geom_point(data = df_il_county_surv_sharp, aes(x=year_var, y=1-year_chf, color=County),
alpha = 0.85) +
  theme(text = element_text(family="Times",size=30),
        plot.title = element_text(size = 30),
        axis.text.x=element_text(size=30),
        panel.background = element_rect(fill = "white", colour = "black"),
        panel.grid.major = element_blank(),
        panel.grid.major.y=element_blank()) +
  scale_color_manual(values=c("darkblue", "darksalmon","goldenrod4", "mediumpurple",
"orchid2")) +
  ylab("Probability CWD (+)") + xlab(" ")

gg_wi_surv <- ggplot() +
  geom_smooth(data = df_wi_county_surv_sharp, aes(x=year_var, y=1-year_chf, color=County),
alpha = 0.85, se = FALSE) +
  #geom_point(data = df_il_county_surv_sharp, aes(x=year_var, y=1-year_chf, color=County),
alpha = 0.85) +
  theme(text = element_text(family="Times",size=30),
        plot.title = element_text(size = 30),
        axis.text.x=element_text(size=30),
        panel.background = element_rect(fill = "white", colour = "black"),
        panel.grid.major = element_blank(),
        panel.grid.major.y=element_blank()) +
  scale_color_manual(values=c("mediumblue", "sienna3", "turquoise", "purple3")) +
  ylab("Probability CWD (+)") + xlab(" ")

sharp_ttl <- df_il_county_surv_sharp %>%
  group_by(year_var) %>%
  summarise( sharp_ttl = sum(sharp))
gg_il_surv_sharp <- ggplot() + geom_line(data = df_il_county_surv_sharp, aes(x=year_var,
y=sharp, color=County), size =1.4) +
  theme(text = element_text(family="Times",size=18),
        plot.title = element_text(size = 18),
        axis.text.x=element_text(size=18),
        panel.background = element_rect(fill = "white", colour = "black"),
        panel.grid.major = element_blank(),
        panel.grid.major.y=element_blank()) +
  scale_color_manual(values=c("darkblue", "darksalmon","goldenrod4", "mediumpurple",
"orchid2")) +
  ylab("Annual Sharpshooting Totals") +
  geom_line(data = sharp_ttl, aes(x=year_var, y=sharp_ttl), size = 2)


grid.arrange(gg_il_surv, gg_wi_surv, ncol=2)



##########################################################################
#
##########################################################################
#
##########################################################################
#
```

```
##########################################################################
#

ggvar <- gg_variable(srf, time = 4)
# For labeling coplot membership
#ggvar$management_var <- paste("edema = ", ggvar$edema, sep = "")

srf$time.interest

levels(ggvar$management_var) <- c("WI: 2001-2007",
                              "WI: 2008-2022",
                              "IL: Hunting",
                              "IL: Other",
                              "IL: Roadkill",
                              "IL: Sharpshooting",
                              "IL: Suspect")
partial_coplot_cwd <- gg_partial_coplot(srf, xvar = "year_var", groups =
ggvar$management_var,
                                   surv_type = "surv",
                                   time = srf$time.interest[6],
                                   show.plots = FALSE)

partial_coplot_cwd <- partial_coplot_cwd[partial_coplot_cwd$group == "WI: 2001-2007" |
                                      partial_coplot_cwd$group == "WI: 2008-2022" |
                                      partial_coplot_cwd$group == "IL: Hunting" |
                                      partial_coplot_cwd$group == "IL: Sharpshooting" ,]

str(partial_coplot_cwd)
gg_time_v_manage_coplot <- ggplot(partial_coplot_cwd, aes(x=year_var, y=1-yhat, col=group))
+
  #geom_smooth(se = FALSE, linetype=c(1,2)) +
  geom_line(size=1.5)+
  labs(x = "Year", y = "Probability CWD (+) at Age=4",
      color = "Management", shape = "Management") +
  scale_color_manual(values=c("darkorange4","darkorange2","midnightblue", "lightslateblue"))
+
  #scale_linetype_manual(values=c("solid","solid","dotted", "dashed"))+
  theme(text = element_text(family="Times",size=36),
       plot.title = element_text(size = 36),
       axis.text.x=element_text(size=36),
       panel.background = element_rect(fill = "white", colour = "black"),
       panel.grid.major = element_blank(),
       panel.grid.major.y=element_blank(), legend.position = "none")



srf$xvar$number_cases_adj_wthin_3_5

partial_coplot_cwd1 <- gg_partial_coplot(srf, xvar = "number_cases_adj_wthin_3_5", groups =
ggvar$state_var,
                                   surv_type = "surv",
                                   time = srf$time.interest[6],
                                   show.plots = FALSE)
```

```
gg_adjcase_lag1_3_coplot <- ggplot(partial_coplot_cwd1,
aes(x=number_cases_adj_wthin_3_5, y=1-yhat, col=group, shape=group)) +
  geom_smooth(se = FALSE) +
  labs(x = "Ttl # of Cases in Adjacent Counties Past 1-3 years", y = "Probability CWD (+) at
Age=4",
       color = "Management", shape = "Management Area") +
  scale_color_manual(values=c("turquoise4","darkorange4"))+
  theme(text = element_text(family="Times",size=30),
        plot.title = element_text(size = 30),
        axis.text.x=element_text(size=30),
        panel.background = element_rect(fill = "white", colour = "black"),
        panel.grid.major = element_blank(),
        panel.grid.major.y=element_blank(),
        legend.title = element_blank())



ggvar$counties_temp <- ggvar$counties
levels(ggvar$counties_temp) <- c(levels(ggvar$counties_temp), "OTHER")
ggvar$counties_temp[ggvar$counties != "BOONE" &
                      ggvar$counties != "MCHENRY" &
                      ggvar$counties != "JODAVIESS" &
                      ggvar$counties != "WINNEBAGO" &
                      ggvar$counties != "ROCK" &
                      ggvar$counties != "GREEN" &
                      ggvar$counties != "WALWORTH" &
                      ggvar$counties != "LAFAYETTE" &
                      ggvar$counties != "STEPHENSON" ] <- "OTHER"

table(is.na(ggvar$counties_temp))
table(ggvar$counties_temp)
ggvar$counties_temp[is.na(ggvar$counties_temp)] <- "OTHER"

partial_coplot_cwd <- gg_partial_coplot(srf, xvar = "number_cases_adj_wthin_3_5", groups =
ggvar$counties_temp,
                                        surv_type = "surv",
                                        time = srf$time.interest[6],
                                        show.plots = FALSE)
class(partial_coplot_cwd)


gg_adjcase_lag3_5_cty_coplot <- ggplot(partial_coplot_cwd[partial_coplot_cwd$group !
="OTHER",],
                                       aes(x=number_cases_adj_wthin_3_5,
                                           y=1-yhat, col=group,
                                           shape=group, alpha=group)) +
  geom_smooth(se = FALSE) +
  labs(x = "Ttl # of Cases in Adjacent Counties Past 1-3 years", y = "Probability CWD (+) at
Age=4",
       color = "Management", shape = "Management Area") +

scale_color_manual(values=c("grey50","grey50","grey50","grey50","aquamarine4","grey50","gr
ey50","goldenrod3","grey50")) +
```

```
  scale_alpha_manual(values=c(0.25,0.25,0.25,0.25,1.00,0.25,0.25,1.00,0.25)) +
  theme(text = element_text(family="Times",size=30),
        plot.title = element_text(size = 30),
        axis.text.x=element_text(size=30),
        panel.background = element_rect(fill = "white", colour = "black"),
        panel.grid.major = element_blank(),
        panel.grid.major.y=element_blank(),
        legend.title = element_blank()) +
  ylim(0,0.15) #+ xlim(0,800)



gg_adjcase_lag3_5_cty_coplot <-
ggplot(partial_coplot_cwd[partial_coplot_cwd$group=="MCHENRY" |
                                            partial_coplot_cwd$group=="WALWORTH" ,],
                                   aes(x=number_cases_adj_wthin_3_5,
                                       y=1-yhat, col=group,
                                       shape=group)) +
  geom_smooth(se = FALSE) +
  labs(x = "Ttl # of Cases in Adjacent Counties Past 3-5 years", y = "Probability CWD (+) at
Age=4",
       color = "Management", shape = "Management Area") +
  scale_color_manual(values=c("aquamarine4","goldenrod3")) +
  theme(text = element_text(family="Times",size=36),
        plot.title = element_text(size = 36),
        axis.text.x=element_text(size=36),
        panel.background = element_rect(fill = "white", colour = "black"),
        panel.grid.major = element_blank(),
        panel.grid.major.y=element_blank(),
        legend.title = element_blank(),
        legend.position = "none") +
  ylim(0.02,0.08) #+ xlim(0,150)



partial_coplot_cwd$State <- partial_coplot_cwd$group
levels(partial_coplot_cwd$State) <- c(levels(partial_coplot_cwd$State), "Wisconsin", "Illinois")
partial_coplot_cwd$State[partial_coplot_cwd$group == "BOONE" |
                         partial_coplot_cwd$group == "WINNEBAGO" |
                         partial_coplot_cwd$group == "JODAVIESS" |
                         partial_coplot_cwd$group == "MCHENRY" |
                         partial_coplot_cwd$group == "STEPHENSON" ] <- "Illinois"
partial_coplot_cwd$State[partial_coplot_cwd$group == "ROCK" |
                         partial_coplot_cwd$group == "GREEN" |
                         partial_coplot_cwd$group == "WALWORTH" |
                         partial_coplot_cwd$group == "LAFAYETTE"] <- "Wisconsin"

gg_adjcase_lag1_3_cty_coplot_WI <-
ggplot(partial_coplot_cwd[partial_coplot_cwd$State=="Wisconsin",],
aes(x=number_cases_adj_wthin_3_5,
                                                             y=1-yhat, col=group,
                                                             shape=group)) +
  geom_smooth(se = FALSE) +
```

```
  labs(x = "Ttl # of Cases in Adjacent Counties Past 1-3 years", y = "Probability CWD (+) at
Age=4",
       color = "Management", shape = "Management Area") +
  scale_color_manual(values=c("turquoise1",
                              "deeppink3","darkorange4",
                              "darkorchid4")) +
  theme(text = element_text(family="Times",size=30),
        plot.title = element_text(size = 30),
        axis.text.x=element_text(size=30),
        panel.background = element_rect(fill = "white", colour = "black"),
        panel.grid.major = element_blank(),
        panel.grid.major.y=element_blank(),
        legend.title = element_blank()) +
  ylim(0,0.20) #+ xlim(0,800)


gg_adjcase_lag1_3_cty_coplot_IL <-
ggplot(partial_coplot_cwd[partial_coplot_cwd$State=="Illinois",],
                                    aes(x=number_cases_adj_wthin_3_5,
                                        y=1-yhat, col=group,
                                        shape=group)) +
  geom_smooth(se = FALSE) +
  labs(x = "Ttl # of Cases in Adjacent Counties Past 1-3 years", y = "Probability CWD (+) at
Age=4",
       color = "Management", shape = "Management Area") +


  scale_color_manual(values=c("darkblue", "darksalmon","goldenrod4", "mediumpurple",
"orchid2")) +
  theme(text = element_text(family="Times",size=30),
        plot.title = element_text(size = 30),
        axis.text.x=element_text(size=30),
        panel.background = element_rect(fill = "white", colour = "black"),
        panel.grid.major = element_blank(),
        panel.grid.major.y=element_blank(),
        legend.title = element_blank()) +
  ylim(0,0.16) +  xlim(0,800)

gg_blank <- ggplot() + theme(text = element_text(family="Times",size=30),
                             plot.title = element_text(size = 30),
                             axis.text.x=element_text(size=30),
                             panel.background = element_rect(fill = "white", colour = "white"),
                             panel.grid.major = element_blank(),
                             panel.grid.major.y=element_blank(),
                             legend.title = element_blank())

partial_coplot_cwd_cull <- gg_partial_coplot(srf, xvar = "all_culled_adj_counties_lag3_5",
groups = ggvar$counties_temp,
                                             surv_type = "surv",
                                             time = srf$time.interest[6],
                                             show.plots = FALSE)

partial_coplot_cwd_cull_temp <- partial_coplot_cwd_cull[partial_coplot_cwd_cull$group ==
"BOONE" |
```

```
                                              partial_coplot_cwd_cull$group == "JODAVIESS" |
                                              partial_coplot_cwd_cull$group == "MCHENRY" |
                                              partial_coplot_cwd_cull$group == "WINNEBAGO" |
                                              partial_coplot_cwd_cull$group == "STEPHENSON" ,]

gg_adjculled_lag3_5_coplot <- ggplot(partial_coplot_cwd_cull_temp,
aes(x=all_culled_adj_counties_lag3_5, y=1-yhat, col=group, shape=group)) +
  geom_smooth(se = FALSE) +
  labs(x = "Ttl Deer Culled in Adjacent Counties Past 3-5 years", y = "Probability CWD (+) at
Age=4",
      color = "Management", shape = "Management") +
  scale_color_manual(values=c("darkblue", "darksalmon","goldenrod4", "mediumpurple",
"orchid2")) +
  theme(text = element_text(family="Times",size=30),
        plot.title = element_text(size = 30),
        axis.text.x=element_text(size=30),
        panel.background = element_rect(fill = "white", colour = "black"),
        panel.grid.major = element_blank(),
        panel.grid.major.y=element_blank(),
        legend.title = element_blank())




lay <- rbind(c(1,1,1,2,2,2),
             c(1,1,1,2,2,2),
             c(3,3,4,4,4,4),
             c(3,3,4,4,4,4))
grid.arrange(gg_surv_by_states,gg_time_v_manage_coplot,gg_adjcase_lag3_5_cty_coplot,
gg_blank, layout_matrix = lay)
setwd("/Users/andrewwhetten/Desktop/")
png("figure_srf_fig2_final.png", width = 5000, height = 4000, res = 200)
grid.arrange( gg_surv_by_states,gg_time_v_manage_coplot,gg_adjcase_lag3_5_cty_coplot,
gg_blank)
dev.off()


lay <- rbind(c(1,1,1,2,2,2),
             c(1,1,1,2,2,2),
             c(1,1,1,3,3,3),
             c(1,1,1,3,3,3))
grid.arrange(gg_surv_by_states,gg_time_v_manage_coplot,gg_adjcase_lag3_5_cty_coplot,
gg_blank, layout_matrix = lay)
setwd("/Users/andrewwhetten/Desktop/")
png("figure_srf_fig2_final_simp.png", width = 4800, height = 3600, res = 200)
grid.arrange(gg_time_v_manage_coplot, gg_adjcase_lag3_5_cty_coplot, gg_blank,
layout_matrix = lay)
dev.off()



grid.arrange(gg_vimp)
grid.arrange( gg_surv_by_states,gg_time_v_manage_coplot,ncol=2)
```

```
grid.arrange( gg_surv_by_states,gg_vimp,gg_time_v_manage_coplot,gg_adjcase_lag1_3_coplo
t)
grid.arrange(gg_il_surv, gg_blank, gg_adjcase_lag1_3_cty_coplot_IL,
gg_adjcase_lag1_3_cty_coplot_WI,
            ncol = 2)
grid.arrange(gg_il_surv, gg_adjcase_lag1_3_cty_coplot_IL, gg_adjcase_lag1_3_cty_coplot_WI,
gg_il_surv_sharp, gg_adjculled_lag3_5_coplot,
            gg_blank, ncol = 3)

##################################################
# Addition Looking at non-border counties ########

# On WI side: Jefferson, Dane, Iowa
# On IL side: LEE, WHITESIDE, ROCKISLAND, LASALLE

jeff_cty_chf <- chf_cov_df[chf_cov_df$counties == "JEFFERSON",]
dane_cty_chf <- chf_cov_df[chf_cov_df$counties == "DANE",]
wauk_cty_chf <- chf_cov_df[chf_cov_df$counties == "WAUKESHA",]

lee_cty_chf <- chf_cov_df[chf_cov_df$counties == "LEE",]
whtsd_cty_chf <- chf_cov_df[chf_cov_df$counties == "WHITESIDE",]
rckisl_cty_chf <- chf_cov_df[chf_cov_df$counties == "ROCKISLAND",]
lasal_cty_chf <- chf_cov_df[chf_cov_df$counties == "LASALLE",]



jeff_cty_chf_sum <- jeff_cty_chf %>%
  group_by(year_var) %>%
  summarise(year_chf = mean(Survival), sharp = sum())
dane_cty_chf_sum <- dane_cty_chf %>%
  group_by(year_var) %>%
  summarise(year_chf = mean(Survival))




lee_cty_chf_sum <- lee_cty_chf %>%
  group_by(year_var) %>%
  summarise(year_chf = mean(Survival), sharp = sum(management_var ==
"SHARPSHOOTING"))
whtsd_cty_chf_sum <- whtsd_cty_chf %>%
  group_by(year_var) %>%
  summarise(year_chf = mean(Survival), sharp = sum(management_var ==
"SHARPSHOOTING"))
lasal_cty_chf_sum <- lasal_cty_chf %>%
  group_by(year_var) %>%
  summarise(year_chf = mean(Survival), sharp = sum(management_var ==
"SHARPSHOOTING"))




# plot(jodaviess_cty_chf_sum$year_var, jodaviess_cty_chf_sum$sharp)
# plot(winnebago_cty_chf_sum$year_var, winnebago_cty_chf_sum$year_chf, type = "b",
```

```
#      col = "blue", ylim = c(0.5,1))
# lines(boone_cty_chf_sum$year_var, boone_cty_chf_sum$year_chf, type = "b", col =
"lightblue")
# lines(mchenry_cty_chf_sum$year_var, mchenry_cty_chf_sum$year_chf, type = "b", col =
"purple")
# lines(jodaviess_cty_chf_sum$year_var, jodaviess_cty_chf_sum$year_chf, type = "b", col =
"darkblue")
# lines(stephenson_cty_chf_sum$year_var, stephenson_cty_chf_sum$year_chf, type = "b", col
= "darkred")
#
# plot(winnebago_cty_chf_sum$year_var, winnebago_cty_chf_sum$sharp, type = "b",
#      col = "blue", ylim = c(0,600))
# lines(boone_cty_chf_sum$year_var, boone_cty_chf_sum$sharp, type = "b", col = "lightblue")
# lines(mchenry_cty_chf_sum$year_var, mchenry_cty_chf_sum$sharp, type = "b", col =
"purple")
# lines(jodaviess_cty_chf_sum$year_var, jodaviess_cty_chf_sum$sharp, type = "b", col =
"darkblue")
# lines(stephenson_cty_chf_sum$year_var, stephenson_cty_chf_sum$sharp, type = "b", col =
"darkred")

df_il_county_surv_sharp <- as.data.frame(rbind(lee_cty_chf_sum,
                                               whtsd_cty_chf_sum,
                                               lasal_cty_chf_sum))
df_wi_county_surv_sharp <- as.data.frame(rbind(jeff_cty_chf_sum[,c(1,2)],
                                               dane_cty_chf_sum))

df_il_county_surv_sharp$County <- rep(c("Lee","Whiteside","LaSalle"),
                                      times=c(18,17,19))
df_wi_county_surv_sharp$County <- rep(c("Jefferson", "Dane"), times = c(21,22))


gg_il_surv_nonb <- ggplot() +
  geom_smooth(data = df_il_county_surv_sharp, aes(x=year_var, y=1-year_chf, color=County),
alpha = 0.85, se = FALSE) +
  #geom_point(data = df_il_county_surv_sharp, aes(x=year_var, y=1-year_chf, color=County),
alpha = 0.85) +
  theme(text = element_text(family="Times",size=30),
        plot.title = element_text(size = 30),
        axis.text.x=element_text(size=30),
        panel.background = element_rect(fill = "white", colour = "black"),
        panel.grid.major = element_blank(),
        panel.grid.major.y=element_blank()) +
  scale_color_manual(values=c("darkblue", "darksalmon","goldenrod4", "mediumpurple",
"orchid2")) +
  ylab("Probability CWD (+)") + xlab(" ")

gg_wi_surv_nonb <- ggplot() +
  geom_smooth(data = df_wi_county_surv_sharp, aes(x=year_var, y=1-year_chf, color=County),
alpha = 0.85, se = FALSE) +
  #geom_point(data = df_il_county_surv_sharp, aes(x=year_var, y=1-year_chf, color=County),
alpha = 0.85) +
  theme(text = element_text(family="Times",size=30),
        plot.title = element_text(size = 30),
        axis.text.x=element_text(size=30),
```

```
       panel.background = element_rect(fill = "white", colour = "black"),
       panel.grid.major = element_blank(),
       panel.grid.major.y=element_blank()) +
  scale_color_manual(values=c("mediumblue", "sienna3", "turquoise", "purple3")) +
  ylab("Probability CWD (+)") + xlab(" ")

grid.arrange(gg_il_surv_nonb, gg_wi_surv_nonb, ncol=2)


sharp_ttl <- df_il_county_surv_sharp %>%
  group_by(year_var) %>%
  summarise( sharp_ttl = sum(sharp))
gg_il_surv_sharp <- ggplot() + geom_line(data = df_il_county_surv_sharp, aes(x=year_var,
y=sharp, color=County), size =1.4) +
  theme(text = element_text(family="Times",size=18),
       plot.title = element_text(size = 18),
       axis.text.x=element_text(size=18),
       panel.background = element_rect(fill = "white", colour = "black"),
       panel.grid.major = element_blank(),
       panel.grid.major.y=element_blank()) +
  scale_color_manual(values=c("darkblue", "darksalmon","goldenrod4", "mediumpurple",
"orchid2")) +
  ylab("Annual Sharpshooting Totals") +
  geom_line(data = sharp_ttl, aes(x=year_var, y=sharp_ttl), size = 2)


grid.arrange(gg_il_surv, gg_wi_surv, ncol=2)

#######################################################
table(ggvar$counties)
ggvar$counties_temp <- ggvar$counties
levels(ggvar$counties_temp) <- c(levels(ggvar$counties_temp), "OTHER")
ggvar$counties_temp[ggvar$counties != "JEFFERSON" &
                    ggvar$counties != "DANE" &
                    ggvar$counties != "LEE" &
                    ggvar$counties != "WHITESIDE" &
                    ggvar$counties != "ROCKISLAND" &
                    ggvar$counties != "LASALLE"  ] <- "OTHER"

table(is.na(ggvar$counties_temp))
table(ggvar$counties_temp)
ggvar$counties_temp[is.na(ggvar$counties_temp)] <- "OTHER"

partial_coplot_cwd <- gg_partial_coplot(srf, xvar = "number_cases_adj_wthin_3_5", groups =
ggvar$counties_temp,
                                 surv_type = "surv",
                                 time = srf$time.interest[6],
                                 show.plots = FALSE)
class(partial_coplot_cwd)

# Conditionally standardize 'value' based on the 'group' column
partial_coplot_cwd <- partial_coplot_cwd %>%
  group_by(group) %>%  # Group the data by the 'group' column
  mutate(adjcase_std = scale(as.numeric(number_cases_adj_wthin_3_5)),
```

```
    yhat_std = scale(1-yhat)) %>% # Standardize the 'value' column within each group
  ungroup() # Ungroup the data frame after applying the transformation



#
# gg_adjcase_lag3_5_cty_coplot <- ggplot(partial_coplot_cwd[partial_coplot_cwd$group !
="OTHER",],
#                                  aes(x=adjcase_std ,
#                                      y=yhat_std, col=group,
#                                      shape=group, alpha=group)) +
#   geom_smooth(se = FALSE) +
#   labs(x = "Ttl # of Cases in Adjacent Counties Past 3-5 years", y = "Probability CWD (+) at
Age=4",
#       color = "Management", shape = "Management Area") +
#   #
scale_color_manual(values=c("grey50","grey50","grey50","grey50","aquamarine4","grey50","gr
ey50","goldenrod3","grey50")) +
#   # scale_alpha_manual(values=c(0.25,0.25,0.25,0.25,1.00,0.25,0.25,1.00,0.25)) +
#   theme(text = element_text(family="Times",size=30),
#        plot.title = element_text(size = 30),
#        axis.text.x=element_text(size=30),
#        panel.background = element_rect(fill = "white", colour = "black"),
#        panel.grid.major = element_blank(),
#        panel.grid.major.y=element_blank(),
#        legend.title = element_blank())
#   # ylim(0,0.15) #+ xlim(0,800)



gg_adjcase_lag3_5_cty_coplot <-
ggplot(partial_coplot_cwd[partial_coplot_cwd$group=="DANE" |
                                                    partial_coplot_cwd$group=="JEFFERSON" ,],
                                aes(x=adjcase_std ,
                                    y=yhat_std,, col=group,
                                    shape=group)) +
  geom_smooth(se = TRUE) +
  labs(x = "Ttl # of Cases in Adjacent Counties Past 3-5 years", y = "Probability CWD (+) at
Age=4",
      color = "Management", shape = "Management Area") +
  scale_color_manual(values=c("aquamarine4","goldenrod3")) +
  theme(text = element_text(family="Times",size=36),
       plot.title = element_text(size = 36),
       axis.text.x=element_text(size=36),
       panel.background = element_rect(fill = "white", colour = "black"),
       panel.grid.major = element_blank(),
       panel.grid.major.y=element_blank(),
       legend.title = element_blank())  +ylab("Relative Cumulative Incidence")#+ xlim(0,150)


gg_adjcase_lag3_5_cty_coplot_il <-
ggplot(partial_coplot_cwd[partial_coplot_cwd$group=="LEE" |
                                                      partial_coplot_cwd$group=="LASALLE" ,],
                                aes(x=adjcase_std ,
```

```
                                          y=yhat_std,, col=group,
                                          shape=group)) +
  geom_smooth(se = TRUE) +
  labs(x = "Ttl # of Cases in Adjacent Counties Past 3-5 years", y = "Probability CWD (+) at
Age=4",
       color = "Management", shape = "Management Area") +
  scale_color_manual(values=c("darkblue", "darksalmon","goldenrod4", "mediumpurple")) +
  theme(text = element_text(family="Times",size=36),
        plot.title = element_text(size = 36),
        axis.text.x=element_text(size=36),
        panel.background = element_rect(fill = "white", colour = "black"),
        panel.grid.major = element_blank(),
        panel.grid.major.y=element_blank(),
        legend.title = element_blank()) +ylab("Relative Cumulative Incidence")
  # ylim(0.0,0.05) #+ xlim(0,150)

grid.arrange(gg_adjcase_lag3_5_cty_coplot_il,gg_adjcase_lag3_5_cty_coplot, ncol=2)



partial_coplot_cwd_harvest <- gg_partial_coplot(srf, xvar = "number_harvest_lag1", groups =
ggvar$counties_temp,
                                       surv_type = "surv",
                                       time = srf$time.interest[6],
                                       show.plots = FALSE)

# Conditionally standardize 'value' based on the 'group' column
partial_coplot_cwd_harv_std <- partial_coplot_cwd_harvest %>%
  group_by(group) %>%  # Group the data by the 'group' column
  mutate(harv_std = scale(number_harvest_lag1),
         yhat_std = scale(1-yhat)) %>% # Standardize the 'value' column within each group
  ungroup() # Ungroup the data frame after applying the transformation


class(partial_coplot_cwd)




gg_harvest_lag1_cty_coplot <-
ggplot(partial_coplot_cwd_harv_std[partial_coplot_cwd_harv_std$group=="DANE" |
                                        partial_coplot_cwd_harv_std$group=="JEFFERSON" ,],
                                      aes(x=harv_std,
                                          y=yhat_std, col=group,
                                          shape=group)) +
  geom_smooth(se = TRUE) +
  labs(x = "Relative Total Havest (Previous Year)", y = "Relative Cumulative Incidence",
       color = "Management", shape = "Management Area") +
  scale_color_manual(values=c("aquamarine4","goldenrod3")) +
  theme(text = element_text(family="Times",size=36),
        plot.title = element_text(size = 36),
        axis.text.x=element_text(size=36),
        panel.background = element_rect(fill = "white", colour = "black"),
        panel.grid.major = element_blank(),
```

```
        panel.grid.major.y=element_blank(),
        legend.title = element_blank()) +ylab("Relative Cumulative Incidence")+
  xlab("Relative Total Harvest")


gg_adjcase_lag3_5_cty_coplot_il <-
ggplot(partial_coplot_cwd_harv_std[partial_coplot_cwd_harv_std$group=="LEE" |

                                               partial_coplot_cwd_harv_std$group=="LASALLE" ,],
                                   aes(x=harv_std,
                                       y=yhat_std, col=group,
                                       shape=group)) +
  geom_smooth(se = TRUE) +
  labs(x = "Ttl # of Cases in Adjacent Counties Past 3-5 years", y = "Probability CWD (+) at
Age=4",
       color = "Management", shape = "Management Area") +
  scale_color_manual(values=c("darkblue", "darksalmon","goldenrod4", "mediumpurple")) +
  theme(text = element_text(family="Times",size=36),
        plot.title = element_text(size = 36),
        axis.text.x=element_text(size=36),
        panel.background = element_rect(fill = "white", colour = "black"),
        panel.grid.major = element_blank(),
        panel.grid.major.y=element_blank(),
        legend.title = element_blank()) +ylab("Relative Cumulative Incidence")+
  xlab("Relative Total Harvest")
  # ylim(0.0,0.05) #+ xlim(0,150)


grid.arrange(gg_adjcase_lag3_5_cty_coplot_il, gg_harvest_lag1_cty_coplot, ncol=2)

gg_adjcase_lag3_5_cty_coplot_il <-
ggplot(partial_coplot_cwd_harv_std[partial_coplot_cwd_harv_std$group=="LEE" |

partial_coplot_cwd_harv_std$group=="LASALLE" ,],
                                   aes(x=number_harvest_lag1,
                                       y=1-yhat, col=group,
                                       shape=group)) +
  geom_smooth(se = FALSE) +
  labs(x = "Ttl # of Cases in Adjacent Counties Past 3-5 years", y = "Probability CWD (+) at
Age=4",
       color = "Management", shape = "Management Area") +
  scale_color_manual(values=c("darkblue", "darksalmon","goldenrod4", "mediumpurple")) +
  theme(text = element_text(family="Times",size=36),
        plot.title = element_text(size = 36),
        axis.text.x=element_text(size=36),
        panel.background = element_rect(fill = "white", colour = "black"),
        panel.grid.major = element_blank(),
        panel.grid.major.y=element_blank(),
        legend.title = element_blank()) +
  ylim(0.0,0.05) #+ xlim(0,150)
```
